# Maximising HIV prevention by balancing the opportunities of today with the promises of tomorrow: a modelling study

**DOI:** 10.1016/S2352-3018(16)30036-4

**Published:** 2016-06-27

**Authors:** Jennifer A Smith, Sarah-Jane Anderson, Kate L Harris, Jessica B McGillen, Edward Lee, Geoff P Garnett, Timothy B Hallett

**Affiliations:** a Imperial College London, London, UK; b Bill & Melinda Gates Foundation, Seattle, WA, USA; c Boston Consulting Group, Boston, MA, USA

## Abstract

**Background:**

Many ways of preventing HIV infection have been proposed and more are being developed. We sought to construct a strategic approach to HIV prevention that would use limited resources to achieve the greatest possible prevention impact through the use of interventions available today and in the coming years.

**Methods:**

We developed a deterministic compartmental model of heterosexual HIV transmission in South Africa and formed assumptions about the costs and effects of a range of interventions, encompassing the further scale-up of existing interventions (promoting condom use, male circumcision, early antiretroviral therapy [ART] initiation for all [including increased HIV testing and counselling activities], and oral pre-exposure prophylaxis [PrEP]), the introduction of new interventions in the medium term (offering intravaginal rings, long-acting injectable antiretroviral drugs) and long term (vaccine, broadly neutralising antibodies [bNAbs]). We examined how available resources could be allocated across these interventions to achieve maximum impact, and assessed how this would be affected by the failure of the interventions to be developed or scaled up.

**Findings:**

If all interventions are available, the optimum mix would place great emphasis on the following: scale-up of male circumcision and early ART initiation with outreach testing, as these are available immediately and assumed to be low cost and highly efficacious; intravaginal rings targeted to sex workers; and vaccines, as these can achieve a large effect if scaled up even if imperfectly efficacious. The optimum mix would rely less on longer term developments, such as long-acting antiretroviral drugs and bNAbs, unless the costs of these reduced. However, if impossible to scale up existing interventions to the extent assumed, emphasis on oral PrEP, intravaginal rings, and long-acting antiretroviral drugs would increase. The long-term effect on the epidemic is most affected by scale-up of existing interventions and the successful development of a vaccine.

**Interpretation:**

With current information, a strategic approach in which limited resources are used to maximise prevention impact would focus on strengthening the scale-up of existing interventions, while pursuing a workable vaccine and developing other approaches that can be used if further scale-up of existing interventions is limited.

**Funding:**

Bill & Melinda Gates Foundation.

## Introduction

The AIDS response has had outstanding success in the development and global scale-up of antiretroviral therapy (ART).^1^ With a few exceptions, there has been less success in reducing the spread of the HIV epidemic.^2^, ^3^, ^4^ However, the sustainability of the response rests upon there being large reductions in new HIV infections in the coming years.^5^

To meet this challenge, prevention interventions have been the focus of much research and development investment; programmes are now in the fortunate position of having a range of interventions to consider, including male condoms, voluntary medical male circumcision (VMMC) services, increased HIV testing and initiation of ART for all diagnosed with HIV (early ART), and oral pre-exposure prophylaxis (PrEP). These interventions need to be prioritised for populations and groups at the greatest risk and who stand to benefit most.^6^, ^7^, ^8^ There is also a growing understanding of the barriers to impact that are faced. These barriers have been articulated as the prevention cascade (Hargreaves and colleagues,^9^ this issue) and include limitations in the supply of, demand for, and adherence to an intervention arising from logistic, structural, and behavioural factors. Arguably, innovative approaches might be able to allow these barriers to increasingly be overcome.

At the same time, there are many advances in the development of new technologies that offer new possible interventions to prevent HIV infections. In the medium term, intravaginal rings and long-acting injectable antiretroviral drugs might be efficacious and acceptable to potential users.^10^, ^11^, ^12^, ^13^, ^14^ In the longer term, effective transfusions of broadly neutralising antibodies (bNAbs), or a vaccine, might become available.^15^, ^16^ These new technological developments offer promise, but it should be remembered that these too will have to overcome the same prevention cascade barriers as other interventions.

For programme planning and for determining investments in research and development, it is crucial to be able to establish priorities among this large portfolio. Usually, the value and probable effect of each intervention is considered separately. However, because they overlap in their functionality and the effect of one intervention affects that of others, a holistic perspective is needed to establish a coherent strategy for HIV prevention that maximises the reduction in HIV incidence and is aligned with information about the epidemic, and the potential risks, costs, and benefits of new developments. To do this, researchers should investigate which configuration of present and future interventions will maximise prevention with available resources, assess the contribution of each component to overall success to inform research and development priorities, and monitor how new information on the success of interventions (in scale-up or further development) informs the balance of the portfolio and the overall effect.

**Research in context**

**Evidence before this study**

Many empirical and modelling studies have investigated the effect and cost-effectiveness of different HIV prevention interventions, singly and in combination, and across different settings. More recently, modelling studies have done these analyses under the constraint of resource allocation—whereby multiple combinations of interventions are examined to understand the best way of spending a fixed budget. We searched PubMed for HIV prevention studies published between Jan 1, 2000, and Dec 31, 2015, with the terms “HIV prevention” AND (“budget allocation” OR “resource allocation”) AND (“model” OR “modeling” OR “modelling”). Our results included a 2013 systematic review of cost-effectiveness modelling studies of PrEP, which found that PrEP has the potential to be a cost-effective addition to HIV prevention programmes in specific settings, particularly when delivered to key populations at highest risk of HIV exposure. We identified 22 abstracts, of which 17 studies met our inclusion criteria of mathematical modelling studies. Most resource allocation models are from a health economics perspective with a short-term outlook, with only a few including a dynamic HIV transmission model (which account for future trends in the epidemic). Of two recent analyses including detailed economic and epidemiological information from Kenya and south India respectively, one shows the additional gains of geographical prioritisation of interventions by local epidemiology, and one shows that as budget levels increase, the optimum intervention strategy is to first increase intervention intensity before scaling up coverage. Other recent studies on new technologies highlight that long-acting PrEP for high-risk women could be cost-effective and that even a medium-efficacy vaccine could have a substantial impact on the epidemic. Resource allocation models are increasingly being developed and used at international and national levels to help guide HIV prevention policy.

**Added value of this study**

We analyse present and future interventions within the framework of optimum resource allocation using a synthesis of all available data. We find that scaling up existing interventions is the most cost-effective way to stem new HIV infections in South Africa, with new technologies able to plug the gaps in cases where the predicted scale-ups are not possible.

**Implications of all the available evidence**

Our findings add to an increasing body of evidence that frontloading HIV prevention investments to maximise the use of interventions which are available now leads to the largest health gains in the long term.

To achieve these aims, we developed a model of the HIV epidemic in South Africa. We incorporated the extent of current intervention scale-up and simulated the cost and effect of a wide portfolio of options for HIV prevention, encompassing the further scale-up of existing interventions and the introduction of interventions focused on new technological developments, to identify optimum allocations of resources.

## Methods

### Overview

We briefly describe the analysis framework, model design, and assumptions about interventions' effects and costs (further details are provided in the appendix). We began by enumerating all the potential interventions (existing and future technologies) that could be scaled up and formed assumptions about the nature of their use (coverage, timing, priority groups) in a range of scenarios (table 1).

We defined three sets of scale-up assumptions that span the range of possible scale-up scenarios for each intervention (except for vaccination, which has only two): the constant scenario assumed no introduction or additional scale-up of prevention interventions; the medium scenario characterised the degree of long-term scale-up that might be anticipated on the basis of current planning; and the maximum scenario determined the fullest possible extent to which those interventions could be deployed. The medium scenario is credible and is based on consultation with a working group of Bill & Melinda Gates Foundation staff for the development of each of these interventions (appendix p 13).

We characterised the optimum set of interventions across a wide range of assumptions for the budget available for HIV prevention. Next, we simulated the effect and evaluated the total cost of implementing the medium scenario from 2016 to 2050. We then explored all possible permutations of the scale-up of the interventions (n=4374, from seven interventions with three possible coverage levels and one intervention with two levels) to determine if other configurations of interventions could, with the same total cost as the medium scenario, achieve a greater impact (defined as more infections averted over the period 2016–50).

We repeated the analysis under alternative sets of assumptions whereby the development of new technologies was not realised, and whereby further scale-up of existing interventions was not possible (table 2).

### Model design

We developed a deterministic compartmental model of heterosexual HIV transmission in South Africa (full description and parameter values are given in Cremin and colleagues^23^ and the appendix). The model represents age, sex, behavioural risk, HIV infection, declining CD4 cell count, ART scale-up, and the eight HIV prevention interventions, and is calibrated to South African demography, age-specific and sex-specific HIV incidence and prevalence, the historical scale-up of ART, circumcision, and patterns of condom use (appendix pp 1–12).^24^, ^25^ We assume that almost all HIV-positive individuals with CD4 counts below 200 cells per μL present for care and receive ART (late ART).

Eight prevention methods were included in the analysis (table 1). Efficacy assumptions for all interventions represent the underlying biological efficacy among people who adhere to the method in question (appendix pp 19–22). For existing methods (male condoms, VMMC, early ART [ie, outreach testing and offering ART to all diagnosed with HIV], and oral PrEP), efficacy estimates were based on current data.^17^, ^18^, ^19^, ^20^, ^21^, ^22^, ^26^, ^27^, ^28^ For new PrEP products (intravaginal rings, long-acting antiretroviral drugs, and bNAbs), the effectiveness of intravaginal rings was based on that observed among the women who were most highly adherent in the Ring Study,^11^ and long-acting antiretroviral drugs and bNAbs were assumed to have a similar effectiveness to oral PrEP but potentially reached a wider part of the population. We included two vaccine formulations: the P5-like vaccine is similar to the pox-protein vaccines in development with 50% efficacy and the idealised vaccine is assumed to have a higher efficacy (70%) and lower attrition rate (5% compared with 10% per year) due to the need for less frequent booster administrations.^16^ In scenarios with vaccination available, we assumed that the P5-like vaccine will be available from 2024, initially for adults and switching to teenagers aged 14 years from 2026. The idealised vaccine will replace the P5-like vaccine from 2030 onwards.

For each intervention, we defined coverage levels for seven population subgroups (female sex workers aged 15–49 years, high-risk women aged 15–29 years, low-risk women aged 15–29 years, high-risk women aged 30–49 years, low-risk women aged 30–49 years, high-risk men aged 15–49 years, low-risk men aged 15–49 years) under each set of scale-up assumptions (appendix pp 15–24). For the PrEP products (oral PrEP, intravaginal rings, long-acting antiretroviral drugs, and bNAbs), the coverage level was the proportion of persons taking PrEP with sufficient adherence such that they benefit fully from the assumed efficacy. A product cannibalism assumption is also incorporated into the PrEP products' coverage levels, such that the introduction of a new product takes some users from existing products but always increases total PrEP coverage (appendix p 14). The relative coverage of each product when implemented in combination is proportional to its coverage in that subgroup when no other PrEP product is available. When any vaccine is introduced, we assumed that oral PrEP, intravaginal rings, long-acting antiretroviral drugs, and bNAbs are scaled back to zero over 5 years from 2035.

Most interventions (condoms, oral PrEP, long-acting antiretroviral drugs, bNAbs) prioritise female sex workers and young women, intravaginal rings prioritise female sex workers, VMMC prioritises young men, and early ART and vaccination are assumed to have uniform coverage by risk, with vaccination primarily targeted to teenagers aged 14 years. All interventions are scaled up linearly over a given time period.

Costs are intended to represent the cost of fully delivering the intervention with the stated scale and efficacy. The rationale for assumptions around fixed and variable costs is given in the appendix. Fixed costs (one-time or recurring annually, but not related to scale) were derived from information about existing product launches. Variable costs (dependent on scale) were based on the population group in question and include the commodity plus a combination of service delivery, testing, laboratory costs, outreach and demand incentives, as appropriate. For all products, there is a step change in the fixed and variable cost when coverage rises above the medium scenario, such that the marginal cost is greater to deliver the intervention at maximum coverage. The total costs for all interventions were scaled up by a factor of 1·43 to represent the 30% of the HIV budget allocated to indirect costs that are not explicitly included here.

Throughout, we refer to each of these interventions with a short-hand label (eg, condoms), but the meaning remains that a full set of activities consistent with increasing the use of condoms as specified in our assumptions (eg, distribution, promotion, supply chain management, social marketing).

### Role of the funding source

A working group of Bill & Melinda Gates Foundation staff (appendix p 13) together with the coauthors contributed to the study design and data collection. With the exception of coauthors, the funder had no further role in data analysis, data interpretation, or writing of the report. The corresponding author had full access to all the data in the study and had final responsibility for the decision to submit for publication.

## Results

The effect of each intervention used in isolation was assessed (appendix p 48). All interventions have the potential to reduce HIV incidence substantially over the period 2016–50. Vaccination has the largest potential impact when scaled up to maximum coverage, followed by long-acting antiretroviral drugs, oral PrEP, bNAbs, and condoms.

**Figure 1:**
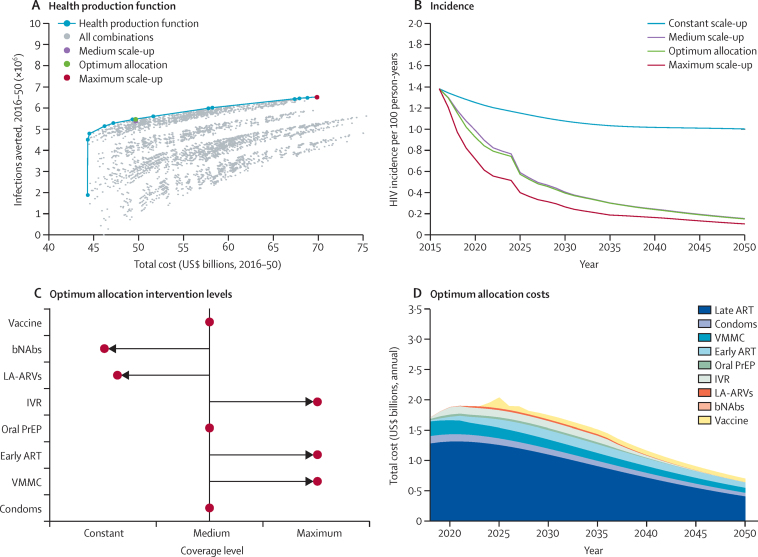
Effect achievable with the full range of interventions (A) The impact and cost of all possible interventions (grey dots), with the frontier highlighted (blue line). The medium scale-up scenario (purple dot), the optimum allocation point on the frontier with the same cost (green dot), and the maximum scale-up scenario (red dot) are highlighted. (B) Trajectory of HIV incidence in 15–49-year-olds, 2016–50, for the constant scale-up, medium scale-up, optimum allocation, and maximum scale-up scenarios. (C) The level of scale-up for each intervention in the optimum allocation scenario. (D) The distribution of costs by intervention type, 2016–50, in the optimum allocation scenario. VMMC=voluntary medical male circumcision. ART=antiretroviral therapy. PrEP=pre-exposure prophylaxis. IVR=intravaginal ring. LA-ARVs=long-acting antiretrovirals. bNAbs=broadly neutralising antibodies.

The cost and effect of all permutations of the scale-up of interventions were examined in the model (figure 1). A frontier can be constructed across these permutations that shows the maximum effect (infections averted with respect to the projected epidemic under the constant assumptions for all interventions) that can be achieved for a given cost. At the low end of the frontier, at which point interventions are scaled up minimally, 1·9 million infections would be averted and the cost of the programme would be US$44 billion ($1·3 billion per year). Most of these costs are attached to spending on late ART for those entering care (84%, data not shown) whereas the effect is produced by increased use of condoms and VMMC (appendix p 45), together with the effect of late ART in reducing transmission. At the high end, at which point all interventions are scaled up to the maximum possible extent, an additional 4·7 million infections are averted at a marginal cost of $26 billion. With increasing available budget, additional interventions are incorporated incrementally (appendix p 45). Condoms, VMMC, early ART, oral PrEP, and a vaccine are implemented at the low end of the frontier, with the inclusion of the intravaginal rings, long-acting antiretroviral drugs, and finally bNAbs, as resources increase.

The medium scenario, where medium coverage is implemented for all interventions, implies a cost of $50 billion over 34 years. The configuration of interventions represented in this scenario is almost on the frontier, signifying that it is approaching the optimum use of resources (figure 1). For the same cost, an alternative configuration of interventions would reduce HIV incidence more rapidly at first and generate 110 000 extra infections averted between 2016 and 2050 (figure 1).

Comparison between the optimum allocation configuration of interventions and the medium coverage level throughout (representing current programmatic aims) shows that increasing coverage of existing interventions (VMMC and early ART) to a greater extent than current projections would be prioritised in the optimum allocation scenario, together with implementing intravaginal rings (figure 1). The scale-up of condoms, oral PrEP, and vaccines in the optimum allocation is consistent with the medium scenario. However, increasing coverage of long-acting antiretroviral drugs is only partly selected and bNAbs are not selected in the optimum configuration of interventions.

In the optimum allocation configuration, the largest amount of resources for prevention interventions is used for VMMC programming (28%; figure 1). If the variable cost of bNAbs was reduced by just 7% it would enter the optimum allocation at low levels and it would require more than 25% reduction in variable cost to be included at higher coverage (appendix p 43). An additional cost of at least $4·5 billion would be required to push vaccination to below medium or maximum coverage in the optimum allocation (appendix p 44).

**Figure 2:**
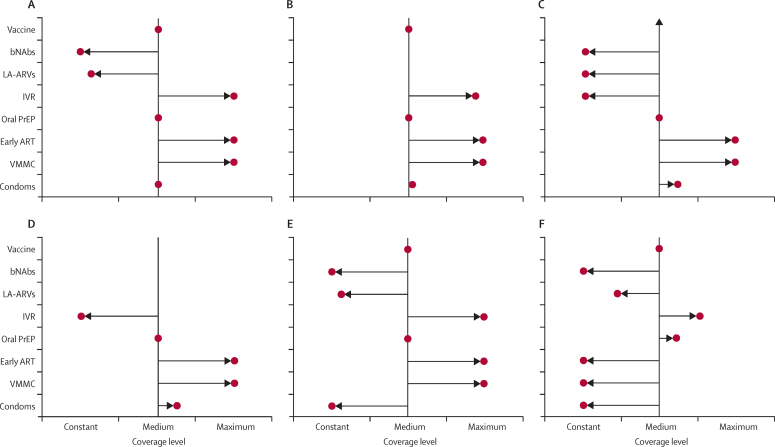
The level of scale-up for each intervention in the optimum allocation scenario, under different assumptions for the availability of interventions (see table 2) Scenario A, all interventions available; scenario B, as A without LA-ARVs or bNAbs; scenario C, as A without vaccine; scenario D, as A without vaccine, LA-ARVs, or bNAbs; scenario E, all interventions available, condom use limited to constant level; scenario F, all interventions available, condom use, VMMC, and early ART limited to constant levels. VMMC=voluntary medical male circumcision. ART=antiretroviral therapy. PrEP=pre-exposure prophylaxis. IVR=intravaginal ring. LA-ARVs=long-acting antiretrovirals. bNAbs=broadly neutralising antibodies.

This analysis was repeated under different assumptions about the availability of interventions (figure 2). The failure for long-term future products to be developed (scenarios B and D in table 2), or the failure for a vaccine to be developed (scenario C and D in table 2), does not substantially affect the overall configuration of the programme in the respective optimum allocation for each. Future products do not attract substantial resources in the optimum allocation scenarios so their loss has little effect (figure 2). However, the financial resources released by the loss of a vaccine are outweighed by those needed to fund the additional ART used if there were no vaccine (given the higher rate of new infections), which also requires the removal of the intravaginal rings from the optimum allocation (figure 2).

The optimum allocation of resources does change if a greater coverage of existing interventions cannot be achieved. If condom programmes are unable to achieve higher coverage than the current level (constant scenario), then the optimum allocation configuration still includes a strong emphasis on VMMC, early ART, intravaginal rings, and vaccination, and maintains a moderate emphasis on oral PrEP (figure 2). If existing interventions (condoms, VMMC, early ART) are all unable to expand further then the optimum configuration of resources includes high levels of oral PrEP and intravaginal rings, plus a greater emphasis on long-acting antiretroviral drugs (figure 2).

**Figure 3:**
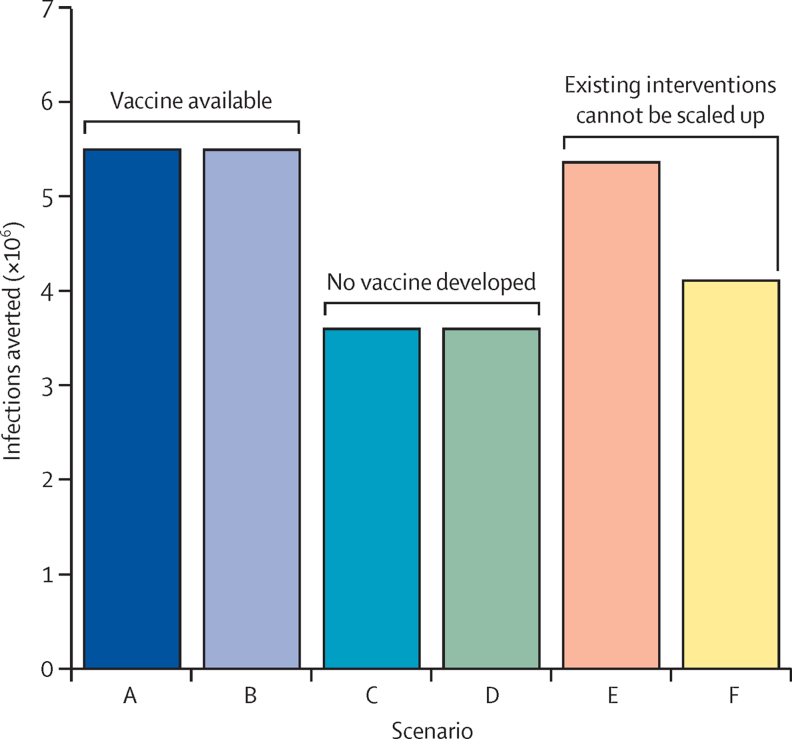
Maximum impact (HIV infections averted 2016–50) achievable with the resources implied in the medium scenario, under different assumptions for the availability of interventions (see table 2) Scenario A, all interventions available; scenario B, as A without long-acting antiretrovirals or broadly neutralising antibodies; scenario C, as A without vaccine; scenario D, as A without vaccine, long-acting antiretrovirals, or broadly neutralising antibodies; scenario E, all interventions available, condom use limited to constant level; scenario F, all interventions available, condom use, voluntary medical male circumcision, and early antiretroviral therapy limited to constant levels.

The biggest single determinant of overall effect is the development of a vaccine (figure 3); the expected number of infections averted is 35% lower without a vaccine. Overall effect is also reduced by up to 23% if the scale-up of condom and other programmes is limited. By contrast, the failure of bNAbs or long-acting antiretroviral drugs to develop has little influence on the overall maximum effect, provided that a vaccine is developed.

A sensitivity analysis where the model was recalibrated for Cross River State, Nigeria, shows a different optimum allocation (for details of calibration see appendix pp 4–6, 46). Cross River State has a concentrated HIV epidemic with lower overall HIV prevalence than South Africa, a smaller group at highest risk driving the epidemic, and very high VMMC coverage.^29^ In this setting, the budget available to allocate across interventions is much lower ($5·6 billion), meaning that fewer interventions are affordable and interventions that target those at highest risk of infection are preferred. In the optimum allocation, condoms, intravaginal rings, and vaccination are all implemented at maximum levels, with all other interventions remaining constant.

## Discussion

Overall, this analysis highlights the need to exploit fully the prevention interventions available today. We find that with a budget consistent with a reasonable set of aims (ie, the budget of the medium scenario), the greatest effect is generated if prevention efforts focus on high coverage of VMMC and early ART programming, use of intravaginal rings particularly by sex workers and, later, achieving high coverage of a vaccine. The single most crucial intervention is the development of a vaccine. However, if it is not possible to scale up existing interventions to the extent envisioned here, the use of PrEP products would become more important to generate the most impact possible.

In this light, a focus on removing the barriers that exist to greater uptake of VMMC, together with early ART, especially, is the priority. An exclusive focus on the development and scaling up of new products, which themselves will have to confront similar barriers, would not generate the greatest effect. However, the widening range of interventions provides opportunities to maintain effect by compensating for failures in other potential interventions.

There are two major limitations to this analysis. First, the representation of product coverage in the model includes three discrete patterns over the different population sub-groups rather than an exploration of all the possible combinations. Although these represent expectations among the authors and the technical group (appendix p 13), this could affect the cost–benefit profile of interventions. For example, products assumed to be targeted to high-risk groups (eg, condom use, PrEP products) might seem more attractive than those that have uniform coverage by risk or age (eg, early ART), and if interventions could be scaled-up differentially by age or risk group, a different combination might have been identified in this analysis as a priority. Furthermore, some interventions could have already reached saturation—for example, condom prevention programmes in hyperendemic settings have been aiming to increase condom use for many years with mixed success.

Second, cost assumptions in the model are not perfectly informed by data. This limitation is inevitable when making projections for scale-up of future interventions. However, we aimed to make consistent assumptions across the interventions for the analysis to be directionally correct; and the total spending in the model for recent years is similar to South Africa HIV programme costs.^30^, ^31^ Estimated condom and VMMC costs are higher in this cost model than the recent South African HIV and TB Investment Case, but the results are broadly consistent with both identified as important priorities.^31^ We do not include research and development costs but these might also be substantial; neither do we include the potential development of drug resistance or the extra treatment costs these could incur.

The analysis focuses on South Africa, but the sensitivity analysis for a lower prevalence setting shows that the results are highly dependent on the epidemic setting. The setting will determine budget level and relative effectiveness of the different interventions according to the size and level of risk across the different population groups. This study complements substantial other modelling work about the allocation of resources in South Africa by including future interventions and taking a long-term perspective.^31^, ^32^ It adds to a growing body of literature on resource allocation for HIV prevention, which highlights the need to account for local epidemiology to maximise the efficiency of HIV prevention planning.^6^, ^33^, ^34^, ^35^, ^36^, ^37^

This analysis represents an interrogation of the information available today, with a view on the evidence of interventions and risk and benefits that is to an extent dependent upon the perspective of the authors. The intention would be that such an analysis would be updated as new information becomes available. A strategic approach in which limited resources are used to maximise prevention effect would focus on strengthening the scale-up of existing interventions, while urgently pursuing a workable vaccine and developing other approaches that can be used if increasing use of existing interventions is limited.

## Figures and Tables

**Table 1:** Summary of intervention assumptions

	**Efficacy**	**Available from (medium and maximum scenarios)**	**Main target group**	**Effective coverage in main target group**	**Fixed cost (US$)**	**Variable cost (US$)**
Condoms	90%^17^	Now	Female sex workers	Constant: 29%; medium: 60%; maximum: 80%	$3·43–8·23 million per partnership type per year	$0·31–0·37 per condom
VMMC	60%^18^, ^19^, ^20^	Now	Young men	Constant: 43%; medium: 80%; maximum: 80%	$5 million launch (high coverage only) plus $27·5–36·3 million per population group per year	$42 per person
Early ART	85%^21^	Now	All	Constant: 0%; medium: 40%; maximum: 60%	$10–11·6 million per year	$275–295 per person per year
Oral PrEP	90%^22^	Now	Female sex workers, high-risk young women	Constant: 0%; medium: 45%; maximum: 80%	$5–15 million launch plus $3·3–11·4 million per year	$170–190 per person per year
IVR	65%^11^	2017	Female sex workers	Constant: 0%; medium: 30%; maximum: 80%	$10 million launch plus $5 million per year	$107–115 per person per year
LA-ARVs	90%	2020	Female sex workers, high-risk young women	Constant: 0%; medium: 50%; maximum: 80%	$10 million launch plus $5 million per year	$180–200 per person per year
bNAbs	90%	2028	Female sex workers, high-risk young women	Constant: 0%; medium: 50%; maximum: 80%	$10 million launch plus $5 million per year	$190–210 per person per year
P5-like vaccine	50%	2024	Teenagers aged 14 years	Constant: 0%; medium and maximum: 70%	$65 million launch plus $5–15 million per year	$40–60 per person per year in first year, $3·5–4·5 per person per year thereafter
Idealised vaccine	70%	2030	Teenagers aged 14 years	Constant: 0%; medium and maximum: 80%	$5 million per year throughout	$50–60 per person per year in first year, $3·5–4·5 per person per year thereafter

Efficacy refers to the protection afforded by perfect use of a product. Effective coverage is the proportion of people who fully adhere to a product such that they benefit from its protection. Full details on coverage, costing, and implementation across all population sub-groups are provided in the appendix. In the Effective coverage in main target group column, the three levels given indicate the assumptions under the constant, medium, and maximum scenarios. VMMC=voluntary medical male circumcision. ART=antiretroviral therapy. PrEP=pre-exposure prophylaxis. IVR=intravaginal ring. LA-ARVs=long-acting antiretrovirals. bNAbs=broadly neutralising antibodies.

**Table 2:** Primary (A) and alternative (B–F) scenarios, where some interventions are removed from consideration or restricted in use

	**Condoms**	**VMMC**	**Early ART**	**Oral PrEP**	**IVR**	**LA-ARVs**	**bNAbs**	**Vaccine**
**All interventions available**								
A	✓	✓	✓	✓	✓	✓	✓	✓
**High-risk technologies not brought to market**								
B	✓	✓	✓	✓	✓	×	×	✓
**No vaccine developed**								
C	✓	✓	✓	✓	✓	✓	✓	×
D	✓	✓	✓	✓	✓	×	×	×
**Condom interventions cannot be scaled up**								
E	*	✓	✓	✓	✓	✓	✓	✓
F	*	✓	✓	*	*	✓	✓	✓

Scenario A, all interventions available; scenario B, as A without LA-ARVs or bNAbs; scenario C, as A without vaccine; scenario D, as A without vaccine, LA-ARVs, or bNAbs; scenario E, all interventions available, condom use limited to constant level; scenario F, all interventions available, condom use, VMMC, and early ART limited to constant levels. VMMC=voluntary medical male circumcision. ART=antiretroviral therapy. PrEP=pre-exposure prophylaxis. IVR=intravaginal ring. LA-ARVs=long-acting antiretrovirals. bNAbs=broadly neutralising antibodies. ✓=intervention is available at all coverage levels. X=intervention is never available.

* Intervention is available only at constant scenario levels.
