# Supplementary material for: Maximising HIV prevention by balancing the opportunities of today with the promises of tomorrow: a modelling study
Source: Lancet HIV. 2016 Jun 27;3(7):e289–96. doi: 10.1016/S2352-3018(16)30036-4 (PMC4935669; doi:10.1016/S2352-3018(16)30036-4)
Supplement: Supplementary appendix [file mmc1.pdf]

THE LANCET HIV

Supplementary appendix

This appendix formed part of the original submission and has been peer reviewed. We post it as supplied by the authors.

Supplement to: Smith JA, Anderson S-J, Harris KL, et al. Maximising HIV prevention by balancing the opportunities of today with the promises of tomorrow: a modelling study. *Lancet HIV* 2016; **3:** 286–96x.

Maximising HIV Prevention - Balancing the Opportunities of Today and the Promises of Tomorrow

Supplementary materials

Model design

The mathematical model is designed to represent heterosexual HIV transmission at the population level in South Africa, a mature generalised HIV epidemic. The model population is divided into compartments that are distinguished by sex, age, infection stage, sexual behaviour and exposure to different types of interventions, with events (e.g. HIV infection, death, ART initiation, etc.) represented as movement between these compartments.[1-3] The model is population-based and deterministic; individuals and partnerships are not explicitly tracked, and the results hold for large populations only.

In the model, the natural history of HIV infection is represented by six consecutive compartments, each relating to a different stage of HIV infection. Individuals becoming infected move from the 'Susceptible' compartment to progress through these six consecutive compartments: 'Acute Infection', a subsequent stage when CD4 count is above 350 cells per microliter, a stage when CD4 count is between 350 and 200 cells per microliter, an 'AIDS (early)' stage, an 'AIDS (advanced)' stage and lastly, an 'AIDS (severe)' stage. Infectiousness of infected individuals varies over the course of infection, with a peak of infectiousness in the 'Acute infection' phase and a period of heightened infectiousness in the 'AIDS (advanced)' phase.[4]

Heterogeneity in sexual behaviour is incorporated in the model by stratifying men and women into risk groups defined by their mean partner change rate. The distribution across these strata and the mean partnership change rates for men and women are estimated through calibrating the model to age-specific HIV prevalence and incidence data from South Africa (see Model Calibration section below).[5]

HIV transmission occurs through heterosexual sexual partnerships. Although these are instantaneous in the model, the influence of variation in duration of partnership is reflected

by specifying the rate of infection through the partnership as a function of the number of sex acts in partnerships per year. That is, short-term partnerships effectively have a low number of sex acts in total, whilst partnerships maintained over a longer time period would have a higher number of sex acts. Those in the higher risk groups tend to form more partnerships, but each of these partnerships is of a shorter duration and comprises fewer sex acts and higher condom use. The probability of HIV transmission per sex act for those in the chronic stage of infection is based on a recent meta-analysis,[6] with the relative infectiousness according to stage of infection based on data from serodiscordant heterosexual couples in sub-Saharan Africa.[4,7]

Figure S1. Representation of HIV natural history and ART initiation

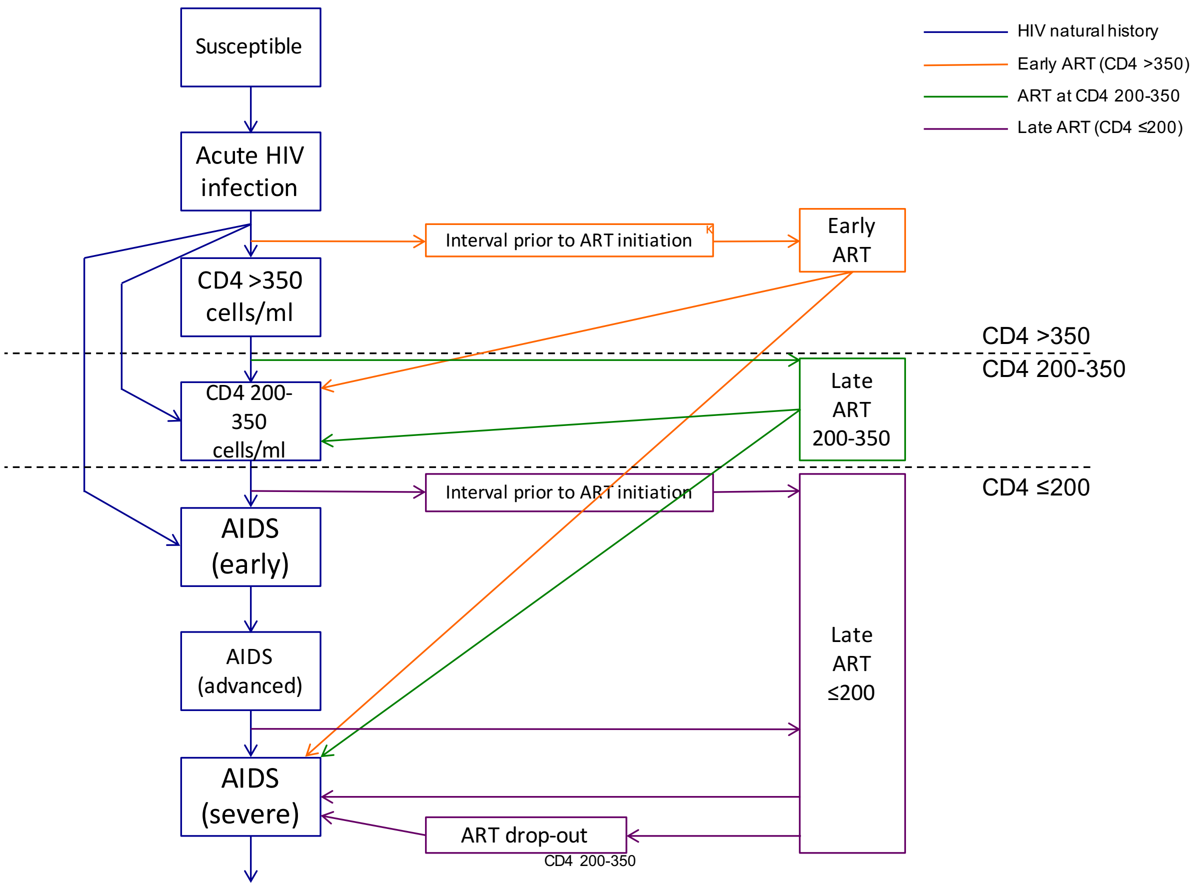

There are four opportunities to initiate ART, specified with different initiation rules. Treatment can be initiated an average of one year after infection (i.e. as soon as infection will be detected on average in an intensive programme), or when an individual's CD4 count drops

below 350, 200, or 50 cells per microliter. In these analyses, ‘Early ART’ refers to the first criterion (i.e. initiated an average of one year after infection) while ‘Late ART’ refers to the latter three (Figure S1). These are used to represent the pattern of actual ART initiation in recent years in South Africa.[8] Individuals initiating ART when their CD4 count drops below 200 do so after a certain waiting time. Due to clinical need, no waiting time is assumed for individuals who are put on treatment when their CD4 drops below 50. Drop outs from the ‘Late ART’ category progress to the AIDS (severe) stage after a period of slightly heightened infectiousness represented by the ‘ART drop-out’ compartment (Figure S1). ART is assumed to extend the survival of treated individuals (depending on whether ART is initiated ‘early’ or ‘late’) while reducing their infectiousness.[7,9]

The impact of condom use is to reduce the chance of transmission in the sex acts in which they are used. There are therefore two parameters specifying the impact of condom use

(1) Efficacy in preventing transmission in a sex act if they are used correctly

(2) Usage in sex acts - proportion of sex acts in a partnership in which they are used (this can vary by partnership type)

Usage changes over time to reflect the increase in condom use in South Africa. Repeated cross-sectional surveys indicate that, reported condom use at last sex increased from 27% in 2002 to 36% in 2012.[5]

The influence of male circumcision is represented by dividing the male population into two categories, circumcised and uncircumcised. Circumcised men are less likely to acquire HIV infection by a fixed multiplicative factor per sex act with HIV-infected women. The probability of transmission of infection per sex act is assumed to be the same from both circumcised and uncircumcised men to women. The proportion of men in the model starting sex that enter the circumcised group corresponds to the proportion of men that are circumcised at birth or during adolescence.[10]

Age-specific fertility and mortality rates over time are taken directly from the ASSA2008 model.[11]

Model calibration

The model was using calibrated using sum of squares for a number of parameters – namely the underlying per sex act transmission probability, risk group sizes and behavioural parameters (Table S1). These were fit to match the model outputs to several data sources:

(1) HIV prevalence of South African adults aged 15+, scaling data from the national antenatal clinic (ANC) survey, as performed by Granich and colleagues.[12,13]

(2) HIV prevalence data from 2002-2012 for adults aged 15-49 from the HSRC 2012 National Survey.[5]

(3) HIV incidence data from 2002-2012 for adults aged 15-49 from the HSRC 2012 National Survey.[5]

(4) Age- and sex-specific prevalence in 2012 for adults aged 15-49 from the HSRC 2012 National Survey.[5]

(5) Age-specific incidence for Kwa-Zulu Natal, 2003-2011, adjusted to match mean incidence to the estimate for South Africa.[14,15]

Sensitivity analyses

Using the same fitting method, we recalibrated the model to Cross River State, Nigeria, to examine the generalizability of the analysis with respect to epidemic setting. Cross River has a more concentrated epidemic than South Africa with high VMMC levels and medium overall prevalence. We fitted the same set of parameters to sex-specific prevalence estimates and ANC trends (adjusted to represent overall female prevalence) only, whilst matching demography and circumcision coverage.[16-19]

Table S1. Fitted model parameters for South Africa and Cross River State, Nigeria

**Parameter**	**Fitted value (South Africa)**	**Fitted value (Cross River, Nigeria)**
HIV transmission probability per sex act	3.52×10^{-4}	1.71×10^{-4}
Degree of assortativity in sexual mixing with respect to risk status	0.0586	0.0999
Degree of assortativity in sexual mixing with respect to age	0.593	0.950
Proportion in [low high FSW] risk group (women)	[0.900 0.0740 0.0260]	[0.656 0.343 0.00119]
Proportion in [low medium high] risk group (men)	[0.817 0.169 0.0143]	[0.518 0.397 0.0848]
Mean partner change rate for low risk group (women)	0.287	0.284
Mean partner change rate for low risk group (men)	1.03	1.24
Multiplicative factor for partner change rate of high risk group (women)	19.2	29.9
Multiplicative factor for partner change rate of FSW (women)	36.9	1490
Multiplicative factor for partner change rate of medium risk group (men)	2.15	1.86
Multiplicative factor for partner change rate of high risk group (men)	108	89.4
Multiplicative factor for 5-year age groups (women): 15-19, 20-24, 25-29, 30-34, 35-39, 40-44, 45-49, 50-54 years	[1.72 2.92 4.09 1.86 1.57 1.17 1.18 0.459]	[1.02 10.0 0.444 0.102 0.989 3.86 9.70 5.35]
Multiplicative factor for 5-year age groups (men): 15-19, 20-24, 25-29, 30-34, 35-39, 40-44, 45-49, 50-54 years	[2.36 4.80 4.38 1.15 2.10 0.866 2.02 0.100]	[0.150 0.721 9.83 0.210 0.137 0.105 0.113 9.10]
Initial condom use by low risk group	3.20×10^{-3}	0.0319

Multiplicative factor for initial condom use by medium risk group (men) and high risk group (women)	1.36	3.57
Multiplicative factor for initial condom use by high risk group (men) and FSW (women)	2.60	15.0

Figure S2. Model fit to South African demography data

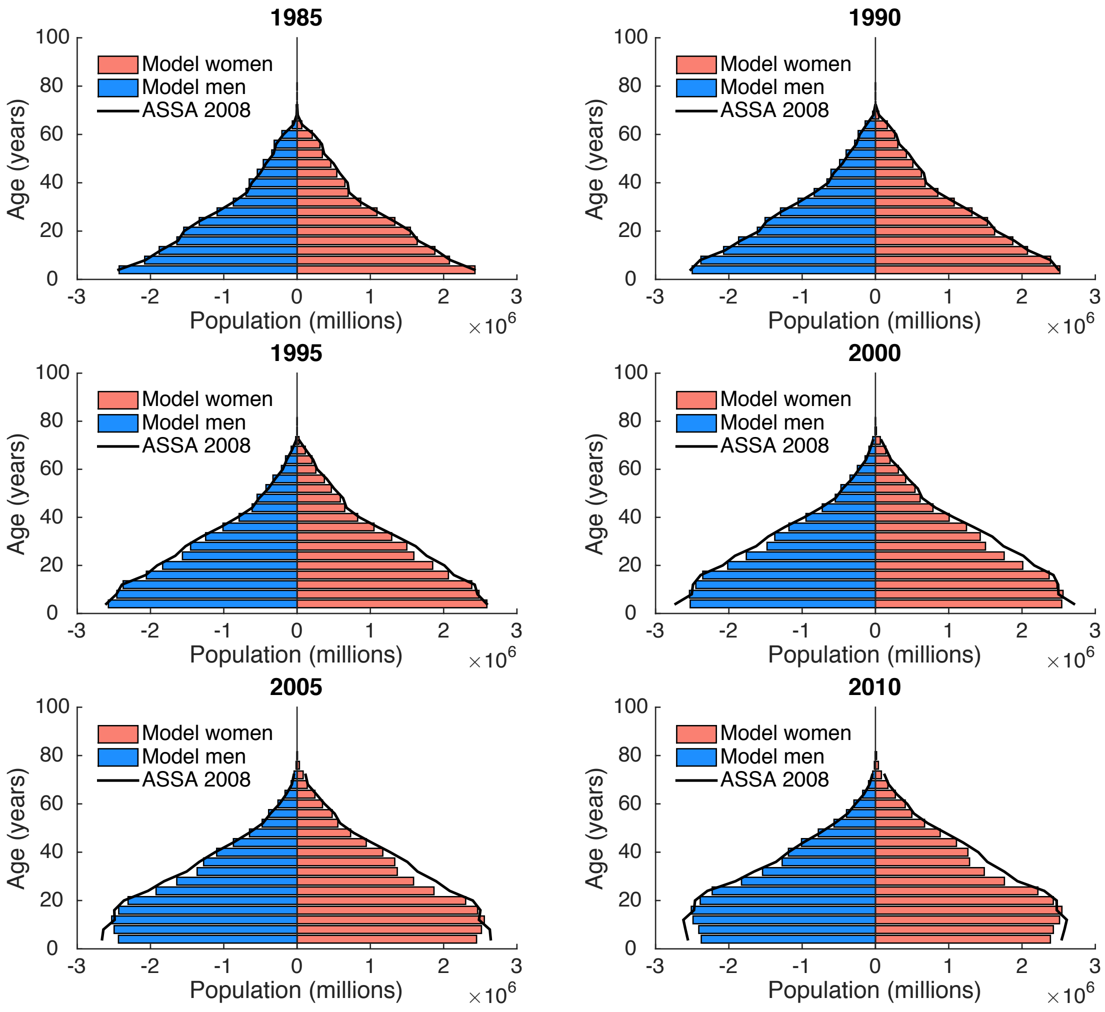

Figure S3. Model calibration to South Africa HIV prevalence and incidence

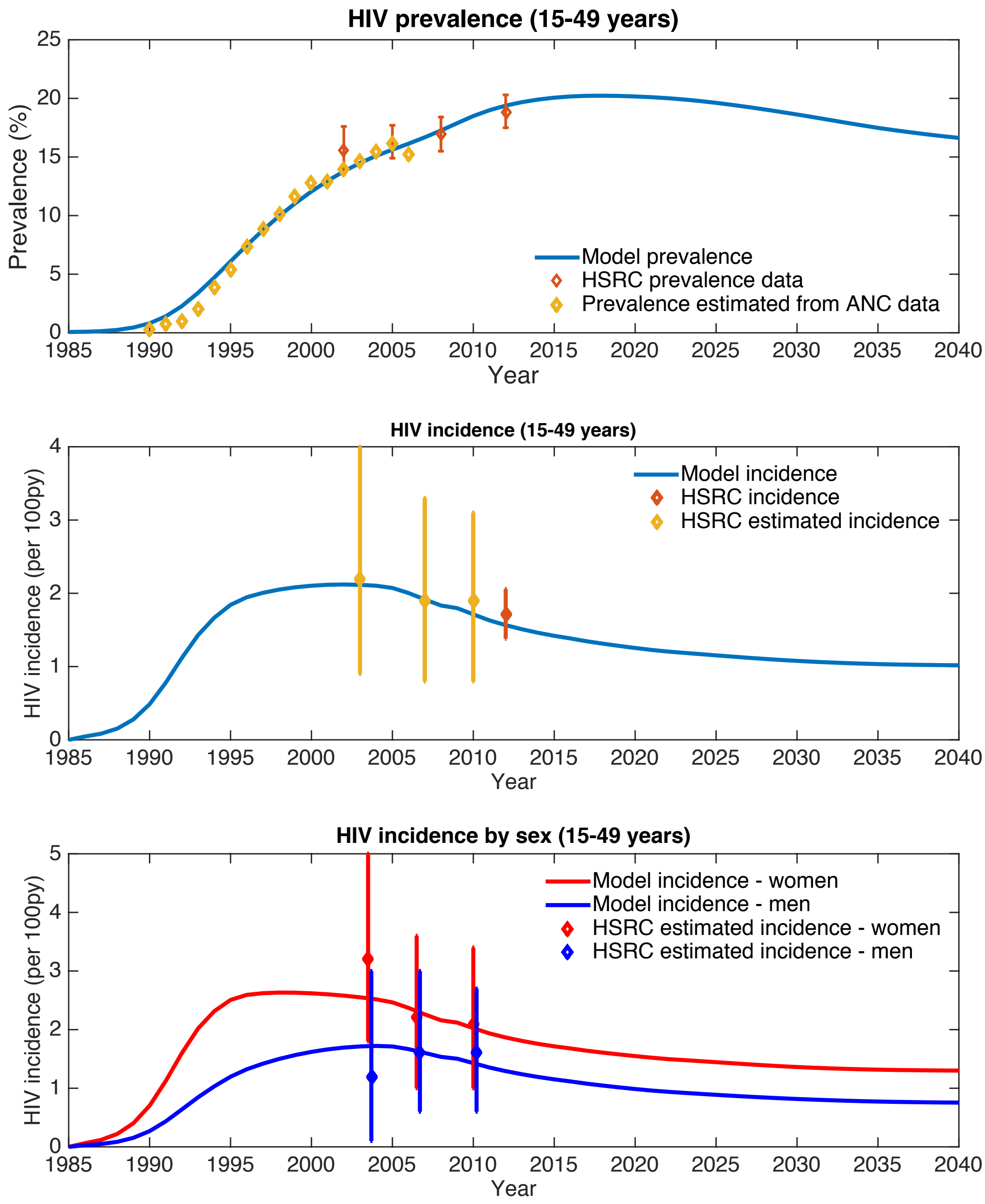

Figure S4. Model fit to age-specific prevalence over time

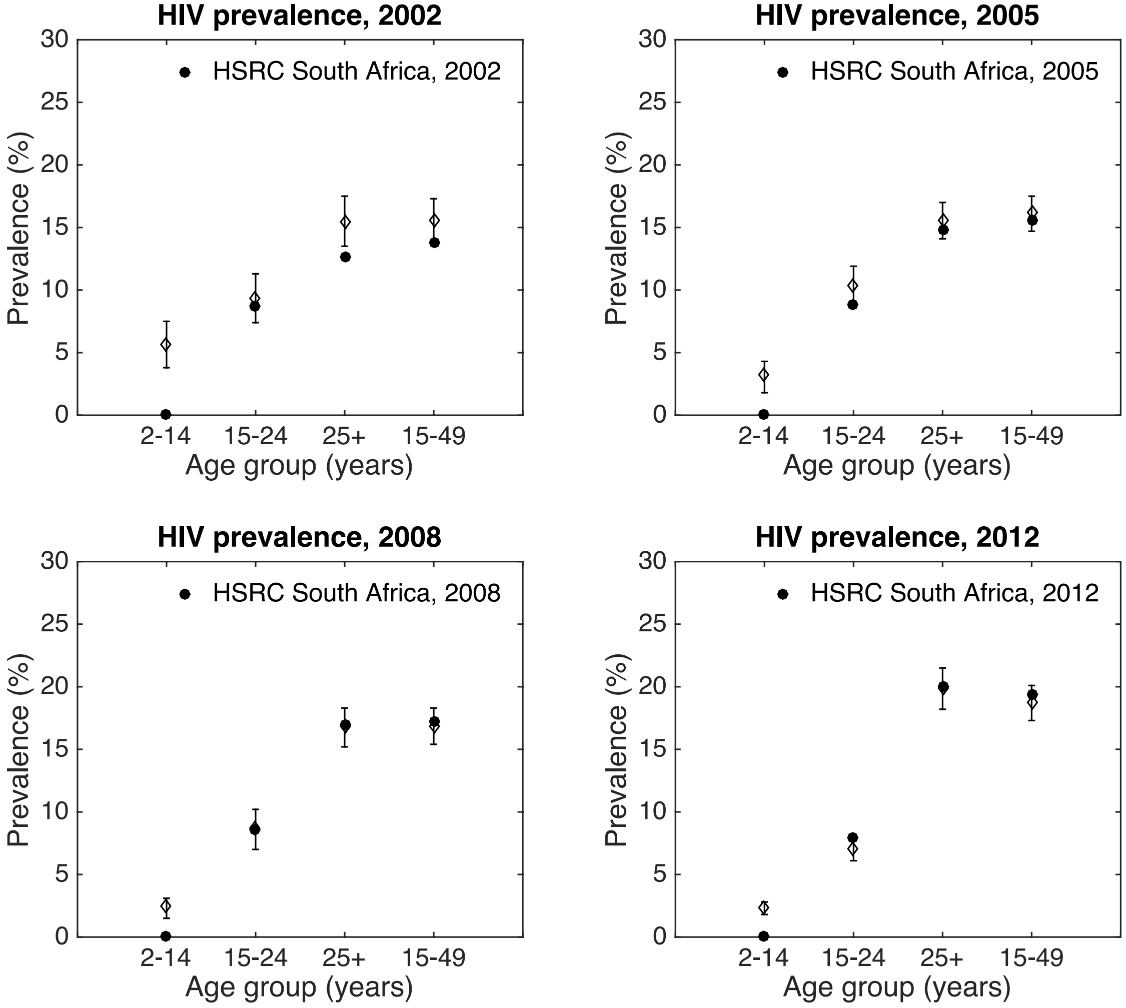

Figure S5. Model fit for age- and sex-specific prevalence in 2012

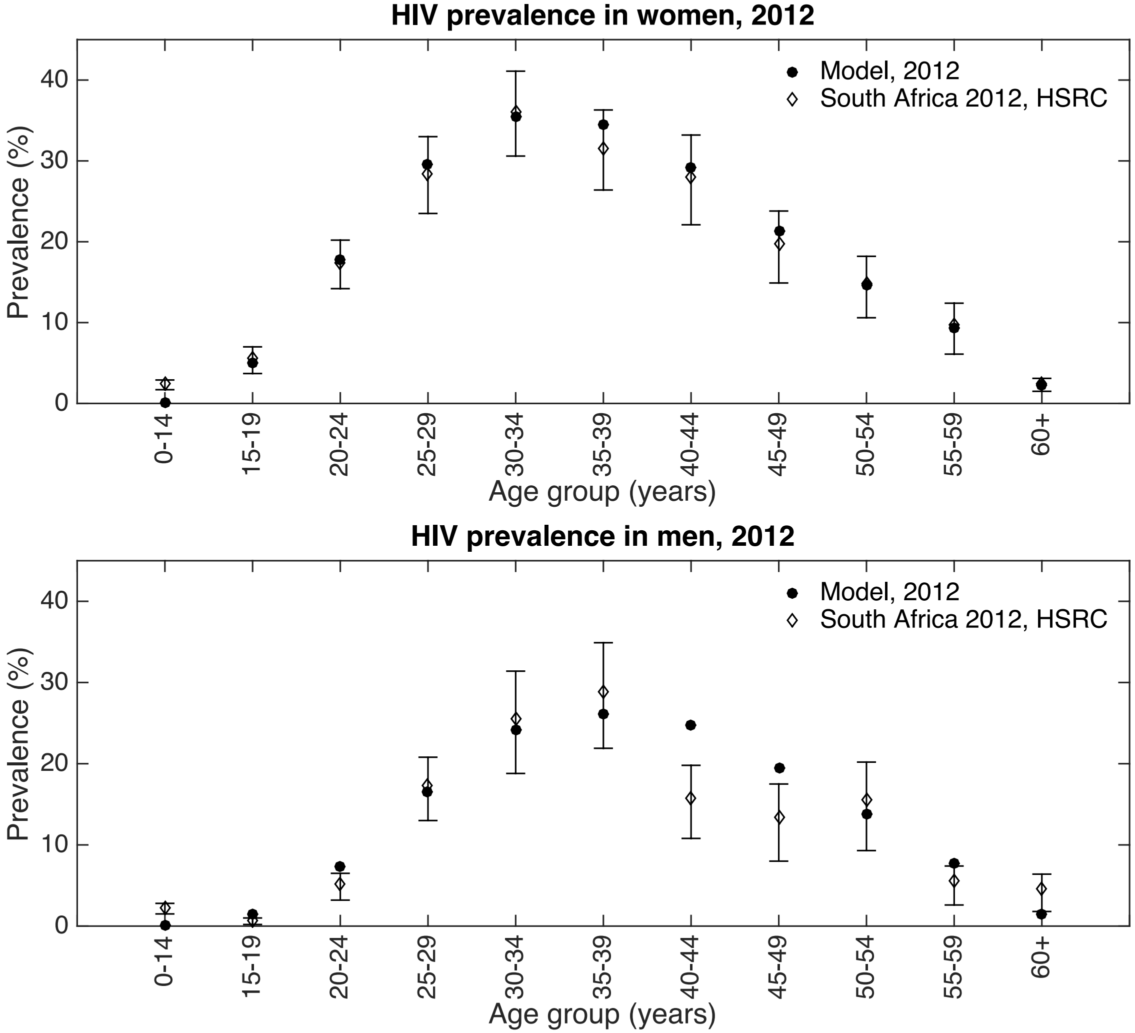

Figure S6. Model fit for age- and sex-specific incidence, 2007

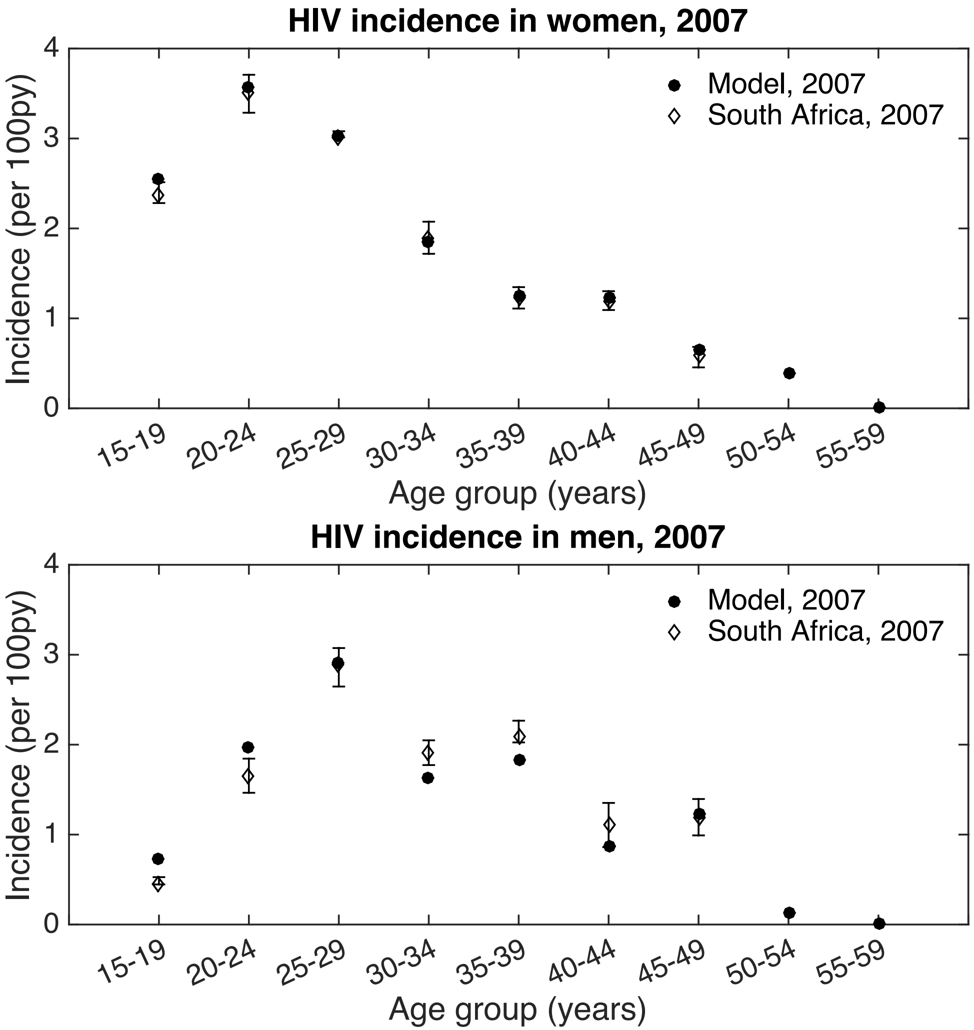

Figure S7. Model fit for number on ART

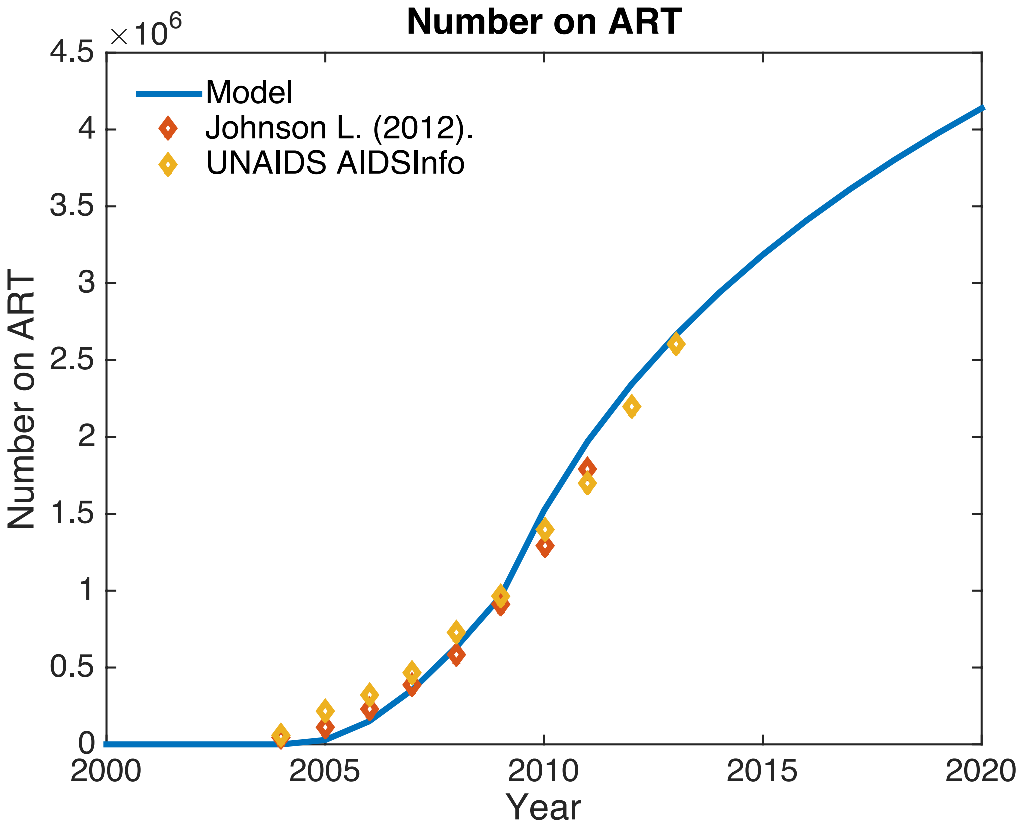

Interventions

The different interventions are incorporated into the model by dividing the population into different strata representing those receiving the intervention and those not receiving it. The different interventions reduce the risk of acquisition of HIV by fixed multiplicative factors per sex act, representing their biological efficacy on transmission from infected to uninfected individuals. The efficacy values are detailed in Tables S4-12. Individuals infected whilst using different products are assumed to have the same infectiousness as others. Effective coverage (representing both usage and adherence) of each intervention can be targeted by age or risk group. Intervention scale-up is linear over a fixed period of time (Tables S4-12), and the target coverage is maintained thereafter.

Table S2. Working group contribution to scenario assumptions

**Name**	**Title**	**Assumptions**
Kate Harris	Program Officer	Fixed Costs, Variable Costs
Gina Dallabetta	Senior Program Officer	Condoms
Maaya Sundaram	Program Officer	Male Circumcision
Geoff Garnett	Deputy Director	Early ART
Lut Van Damme	Senior Program Officer	Oral PrEP, Long acting ARVs, Intravaginal Rings
Silvija Staprans	Senior Program Officer	bNAbs, Vaccines

PrEP products: product cannibalism

When more than one PrEP product (oral PrEP, IVR, LA ARVs, bNAbs) is available, newer products may cannibalize users of existing products. Coverage of each product is determined by (1) the number of products in use; and (2) the univariate coverage of each product as a proxy for user preference (Tables S7-10). Maximum PrEP coverage is capped at 90%. For each population sub-group, when an additional PrEP product becomes available the total PrEP coverage is capped at the value given in Table S3, and the coverage for each product is adjusted such that the relative coverage across PrEP products matches their respective coverage levels when implemented individually.

Table S3. Total coverage level of PrEP products (oral PrEP, IVR, LA-ARVs, bNAbs)

	Total PrEP coverage (%) with					
Population group	Two interventions		Three interventions		Four interventions	
	Medium effective coverage	Maximum effective coverage	Medium effective coverage	Maximum effective coverage	Medium effective coverage	Maximum effective coverage
Female sex workers	55	90	63	90	70	90
High-risk women, 15-30 years	21	48	28	55	34	60
Low-risk women, 15-30 years	4	12	4	16	n/a	20
High-risk women, 30-49 years	10	13	10	16	n/a	20
Low-risk women, 30-49 years	4	11	4	15	n/a	20
High-risk men, 15-49 years	4	13	4	14	n/a	n/a
Low-risk men, 15-49 years	4	11	n/a	11	n/a	n/a

Coverage totals for multiple product use are based on discussions with the Working Group (Table S2) and designed to reflect assumed user adherence and preferences.

Table S4. Intervention assumptions for male condoms.
Partnership types are described by both participants; in case of conflict, we use the higher value for condom use from the two. Where a range of values are given, coverage increases from the lower to the higher value in the period 1995-2008 due to behaviour change.

<table>
<tr><th rowspan="2">Partnership type</th><th rowspan="2">Efficacy</th><th rowspan="2">Current coverage</th><th colspan="3">Effective coverage
(number of sex acts with condom / total sex acts)</th><th rowspan="2">Coverage increase starts</th><th rowspan="2">Duration for coverage change</th></tr>
<tr><th>Constant</th><th>Medium</th><th>Maximum</th></tr>
<tr><td>Female sex worker <> Client</td><td rowspan="7">90%[20]</td><td rowspan="7">Informed by data and in model calibration</td><td>0.8 - 29%</td><td>60%</td><td>80%</td><td rowspan="7">2016</td><td rowspan="7">5</td></tr>
<tr><td>High-risk women, 15-30 years <> Anyone</td><td>0.4 - 28%</td><td>0.4 - 28%</td><td>60%</td></tr>
<tr><td>Low-risk women, 15-30 years <> Anyone</td><td>0.4 - 28%</td><td>0.4 - 28%</td><td>40%</td></tr>
<tr><td>Women, 30+ years <> Anyone</td><td>0.4 - 28%</td><td>0.4 - 28%</td><td>0.4 - 28%</td></tr>
<tr><td>High-risk men <> Low-risk women</td><td>0.4 - 28%</td><td>0.4 - 28%</td><td>0.4 - 28%</td></tr>
<tr><td>High-risk men <> High-risk women</td><td>0.4 - 28%</td><td>0.4 - 28%</td><td>70%</td></tr>
<tr><td>Low-risk men <> Anyone</td><td>0.2 - 14%</td><td>0.2 - 14%</td><td>20%</td></tr>
</table>

Low-risk men <> Low-risk women (serodiscordant couples)			0.2 - 14%	0.2 - 14%	80%		

The condom efficacy estimate represents consistent users.[20] This is higher than the most recent Cochrane review (~80% effectiveness) but that estimate is covers all condom users rather than consistent users only.[21]

Table S5. Intervention assumptions for voluntary medical male circumcision (VMMC).
The model simulates the minimum sufficient circumcision operations required in order to induce at least these levels of coverage.

<table>
<tr><th rowspan="2">Population group</th><th rowspan="2">Efficacy</th><th rowspan="2">Current coverage</th><th colspan="3">Effective coverage
(number of circumcised men / number of men)</th><th rowspan="2">Coverage increase starts</th><th rowspan="2">Duration for coverage change</th></tr>
<tr><th>Constant</th><th>Medium</th><th>Maximum</th></tr>
<tr><td>High-risk men, 15-30 years</td><td rowspan="4">60%[22-24]</td><td rowspan="4">Informed by data and in model calibration</td><td rowspan="2">43%</td><td>80%</td><td>80%</td><td rowspan="4">Now</td><td rowspan="4">5</td></tr>
<tr><td>Low-risk men, 15-30 years</td><td>70%</td><td>80%</td></tr>
<tr><td>High-risk men, 30-49 years</td><td rowspan="2">10%</td><td>15%</td><td>15%</td></tr>
<tr><td>Low-risk men, 30-49 years</td><td>15%</td><td>15%</td></tr>
</table>

Table S6 Intervention assumptions for early ART
Early ART is defined as >350 CD4 cells per ml[3].

Population group	Efficacy	Current coverage	Effective coverage (number of people that can receive early ART early / total number of HIV-positive people)			Coverage increase starts	Duration for coverage change
			Constant	Medium	Maximum		
Female sex workers	85%[9]	0%	0%	40%	60%	2016	2
High-risk women, 15-30 years			0%	40%	40%		
Low-risk women, 15-30 years			0%	40%	40%		
High-risk women, 30-49 years			0%	40%	40%		
Low-risk women, 30-49 years			0%	40%	40%		
High-risk men, 15-49 years			0%	40%	40%		
Low-risk men, 15-49 years			0%	40%	40%		

The efficacy estimate includes the assumption that up to 90% of users will be virally suppressed.

Table S7. Intervention assumptions for oral PrEP
Coverage is defined as the coverage of "good users" benefiting from the efficacy values, which takes into account levels of adherence. Wastage of PrEP through temporary provision to bad users is assumed to be incorporated into the average unit costs of PrEP.

Population group	Efficacy	Current coverage	Effective coverage (number of adherent users / total number of people)			Available from	Duration for coverage change
			Constant	Medium	Maximum		
Female sex workers	90%[25]	0%	0%	45%	80%	2016	2
High-risk women, 15-30 years				15%	30%		
Low-risk women, 15-30 years				0%	10%		
High-risk women, 30-49 years				0%	10%		
Low-risk women, 30-49 years				0%	10%		
High-risk men, 15-49 years				0%	10%		
Low-risk men, 15-49 years				0%	10%		

Oral PrEP efficacy is the per-sex act value for good adherers, derived from analysis of individuals in the Partners PrEP study with tenofovir concentrations >40ng/mL.[25] The proportion of each sub-group of the population assumed to be "good adherers" is incorporated into the coverage assumption.

Table S8. Intervention assumptions for the intravaginal ring.
Coverage is defined as the coverage of "good users" benefiting from the efficacy values, which takes into account levels of adherence. Wastage of IVR through temporary provision to bad users is assumed to be incorporated into the average unit cost. We assume that some of this coverage is cannibalistic on the oral PrEP coverage levels.

Population group	Efficacy	Current coverage	Effective coverage (number of adherent users / total number of people)			Available from	Duration for coverage change
			Constant	Medium	Maximum		
Female sex workers	65%[26]	0%	0%	30%	80%	2017	2
High-risk women, 15-30 years				0%	4%		
Low-risk women, 15-30 years				0%	4%		
High-risk women, 30-49 years				10%	10%		
Low-risk women, 30-49 years				2%	10%		
High-risk men, 15-49 years				0%	0%		
Low-risk men, 15-49 years				0%	0%		

We assume 65% efficacy for the intravaginal ring, in line with the effectiveness reported by Nel and colleagues in the strata of users with the highest adherence (combined measure of drug in blood plasma and returned rings) in The Ring Study.[26] We assume no effective coverage among 15-30 year-old women in the 'Medium' scenario, and very low levels in the 'Maximum' scenario to represent low adherence in this young age group. There is no cost attached to zero usage, as we assume that these women will not return for product refills.

Table S9. Intervention assumptions for long-acting injectable ARVs.
Coverage is defined as the coverage of “good users” benefiting from the efficacy values, which takes into account levels of adherence. Wastage of LA ARVs through temporary provision to bad users is assumed to be incorporated into the average unit cost. We assume that some of this coverage is cannibalistic on other forms of PrEP.

Population group	Efficacy	Current coverage	Effective coverage (number of adherent users / total number of people)			Available from	Duration for coverage change
			Constant	Medium	Maximum		
Female sex workers	90%	0%	0%	50%	80%	2020	2
High-risk women, 15-30 years				15%	30%		
Low-risk women, 15-30 years				4%	10%		
High-risk women, 30-49 years				4%	10%		
Low-risk women, 30-49 years				4%	10%		
High-risk men, 15-49 years				4%	10%		
Low-risk men, 15-49 years				4%	10%		

We have matched the long-acting injectable PreP efficacy to that of oral PrEP, based on the assumption that the biological protection would be similar. There are limited studies to support this; raltegravir provides similar protection to oral PrEP in a mouse model and results from the LATTE 2 trial at CROI 2016 showed that when used as therapy, a combination of long-acting injectable cabotegravir and rilpivirine was equivalent to an oral regimen at maintaining viral suppression.[27,28]

Table S10. Intervention assumptions for broadly neutralising antibodies.
Coverage is defined as the coverage of "good users" benefiting from the efficacy values, which takes into account levels of adherence. Wastage of bNAbs through temporary provision to bad users is assumed to be incorporated into the average unit cost. We assume that some of this coverage is cannibalistic on other forms of PrEP.

Population group	Efficacy	Current coverage	Effective coverage (number of adherent users / total number of people)			Available from	Duration for coverage change
			Constant	Medium	Maximum		
Female sex workers	90%	0%	0%	50%	80%	2028	5
High-risk women, 15-30 years				15%	30%		
Low-risk women, 15-30 years				4%	10%		
High-risk women, 30-49 years				4%	10%		
Low-risk women, 30-49 years				4%	10%		
High-risk men, 15-49 years				4%	10%		
Low-risk men, 15-49 years				4%	10%		

The efficacy of bNAbs is unknown; again in the absence of robust data we have matched it to that of oral PrEP. One study on antibody 3BNC117 showed a 0.8-2.5 log reduction in incidence for 28 days, for which the upper estimate could correspond to a 90% reduction in per-sex act transmission risk.[29,30] We have not addressed the potential emergence of viral resistance to bNAbs but assume that any future formulation brought to market would limit this risk as far as possible.

Table S11. Intervention assumptions for P5-like vaccine.

Partnership type	Efficacy	Current Coverage	Effective coverage (number of people vaccinated / total number of people)		Available from	Duration for coverage change
			Constant	Medium / Maximum		
All population groups	50%	0%	0%	Continuously (2024-2026): Annual cohorts of 18-year-olds: 40% vaccinated<br>One-off campaign (2024): 19-45 year-olds: 20%<br>Continuously (after 2026): Annual cohorts of 14-year-olds: 70% vaccinated<br>One-off campaign (2026): 15-17 year-olds: 40%<br>Attrition of 10% per year of persons ceasing to return for boosting.	2024	5

Table S12. Intervention assumptions for idealised vaccine.

Partnership type	Efficacy	Current Coverage	Effective coverage (number of people vaccinated / total number of people)		Available from	Duration for coverage change
			Constant	Medium / Maximum		
All population groups	70%	0%	0%	Continuously (after 2030): Annual cohorts of 14-year-olds: 80% vaccinated<br>One-off campaign (2030): 15-45 year-olds: 40%<br>Attrition of 5% per year of persons ceasing to return for boosting	2024	5

We assume that a moderately effective (P5-like, 50%) vaccine is introduced in 2024 and replaced by a more effective version (70%) from 2030. For the former, initially 24 year-olds are vaccinated (with a catch-up campaign for 19-45 year-olds) but from 2026 the target age drops to 14 year-olds (with a catch-up campaign for 15-17 year-olds), reflecting the probability that regulatory approval for adolescents will be granted later than for adults. The lower efficacy vaccine is based on the pox protein vaccines that are in development by the P5 Partnership (these use the data around immune correlates from the RV144 vaccine study together with different adjuvants to try and improve effectiveness & longevity).[31] We assume a 50% effective vaccine would be licensed, but that continuing vaccine research could lead to further improvements, hence the 70% effective vaccine becoming available in 2030. This latter assumption is purely hypothetical.

Cost model assumptions

Scope:

For all interventions, total costs include fixed and variable costs to estimate the comprehensive cost of increasing coverage to additional populations from a provider prospective.

Fixed costs include one-time and general population investments needed to launch and sustain intervention, agnostic of coverage reached. Examples of fixed costs are mass media campaigns to promote interventions and launch planning costs for new interventions.

Variable costs include all costs needed to reach the individual at various coverage levels. Variable costs (dependent on scale) are based on the population group in question and include the commodity plus a combination of service delivery, testing, laboratory costs, outreach and demand incentives, as appropriate. Outreach and demand incentives include estimated investments needed to create awareness and increase the accessibility to a given service. Examples of investments include community health worker outreach visits to female sex workers (FSWs) to create awareness of new and existing HIV prevention options and transport vouchers for target populations aimed at increasing their demand for preventative care by reducing their out-of-pocket costs, a potential barrier to service.

Methods:

Economic costs are used for all interventions. Fixed costs are evenly distributed across all population groups for a given intervention, agnostic of scale. For new interventions (namely oral PrEP, IVR, LA-ARVs, bNABs, and the HIV vaccine) fixed costs include launch planning costs. These estimates are sourced from the LIST Uptake Costing Tool, an Excel-based tool designed by Dalberg Consulting for BMGF to approximate launch costs for new products. The scope of the launch costs include market management, policy guidance, WHO pre-qualification, regional and country policy consultations, demand creation, and delivery capacity. Costs are based on historical estimates of previous product launches. All mass media costs with the exception of VMMC were estimated by BMGF based on discussions with current providers of HIV prevention programs. Variable costs are based on unit costs that may vary with coverage levels, recognizing the impact of scale on marginal costs. Unit cost estimates are based on a combination of costing literature and internal BMGF estimates, the latter especially for products still under development. All costs are discounted at 3% per year.

Table S13. Cost assumptions for male condoms.

Partnership type	Coverage level	Fixed costs	Variable costs
Female sex worker <> Client	Constant	0	$0.31 per condom
	Medium	$3.43M / year<br>- Mass media (for clients): $1.43M/year<br>- Programme management: $2M/year	$0.31 per condom
	Maximum	$8.23M / year<br>- Mass media (for clients): $1.43M/year<br>- Programme management: $2M/year<br>- Outreach: $4.8M/year	$0.37 per condom
High-risk women, 15-30 years <> Anyone	Constant, Medium	0	$0.31 per condom
	Maximum	$3.43M / year	$0.37 per condom
Low-risk women, 15-30 years <> Anyone	Constant, Medium	0	$0.31 per condom
	Maximum	$3.43M / year	$0.37 per condom
Women, 30+ years <> Anyone	Constant, Medium	0	$0.31 per condom
	Maximum	$3.43M / year	$0.37 per condom
High-risk men <> Anyone	Constant, Medium	0	$0.31 per condom
	Maximum	$3.43M / year	$0.37 per condom
Low-risk men <> Anyone	Constant, Medium	0	$0.31 per condom
	Maximum	$3.43M / year	$0.37 per condom
	Constant, Medium	0	$0.31 per condom

Low-risk men <> Low-risk women (serodiscordant couples)	Maximum	$3.43M / year	$0.37 per condom

The fixed cost of condom use is the same for every partnership type and includes $10M for mass media ($1.43M per partnership type) plus $2M per year for NGO program set up and management costs. These costs were estimated by BMGF based on discussions with current providers of HIV prevention programs. The variable cost per condom allows each partnership type to accumulate differential variable costs according to coital frequency and levels of condom use. The per condom cost for constant and medium coverage levels is based on $0.29/condom (the current cost paid by the South African government for regular condoms including distribution) plus $0.02/condom for lubrication (based on $0.10 per sachet, used in 20% of sex acts). At maximum coverage levels, we use a mean of $0.29/condom and $0.40/condom, which allows 50% of condoms to be higher-priced varieties, plus the $0.02/condom cost for lubrication. Outreach costs (for sex workers only) assume monthly visits to increase awareness on condoms, PrEP, vaginal rings, and to distribute condoms and lube. We assume a 1:1 model between the three interventions, so $40 per FSW is shared by 3 programs ($13 each). We have converted this into an annual cost by multiplying by the number of sex workers and added this figure to fixed rather than variable costs due to the model's structure for condom use. These costs are based on published literature of the Avahan program.[32] These costs do not include any additional cost for wastage.

Table S14. Cost assumptions for VMMC

Population group	Coverage level	Fixed costs	Variable costs
High-risk men, 15-30 years	Constant, Medium	$27.5M / year<br>- Mass demand generation: $3.9M/year<br>- Programme management: $7.9M/year<br>- Maintenance / infrastructure: $3.2M/year<br>- Capital: $0.9M/year<br>- Salaries: $11.6/year	$42/person<br>- Commodity (kits): $20/person<br>- Labs: $6/person<br>- Demand generation: $9/person<br>- Salary incentives: $7/person
	Maximum	$36.3M / year +<br>- Mass demand generation: $5.1M/year<br>- Programme management: $10.4M/year<br>- Maintenance / infrastructure: $4.3M/year<br>- Capital: $1.2M/year<br>- Salaries: $15.3M/year<br>$1.25M one time<br>- Supply chain and M&E system investments	
Low-risk men, 15-30 years	Constant, Medium	$27.5M / year	
	Maximum	$36.3M / year +<br>$1.25M one time	
High-risk men, 30-49 years	Constant, Medium	$27.5M / year	
	Maximum	$36.3M / year +<br>$1.25M one time	
Low-risk men, 30-49 years	Constant, Medium	$27.5M / year	
	Maximum	$36.3M / year +<br>$1.25M one time	

The coverage target is estimated to reach 1.85M or 4.3M men, for Medium and Maximum coverage levels respectively.
Fixed costs are based on estimates from 2013 CareWorks financial data of a VMMC program in South Africa. All mass demand generation channels (field mobilisation, technology, bathroom marketing, corporate) average $19.50/person/year or $84M for the total population (Maximum). Field mobilization is the most cost-effective option, at $8.40/person/year = $36M, or $9M per each of the 4 sub-groups for all 4.3M people. We assume only field mobilization will be deployed. All other fixed costs are sourced from a costing study of a VMMC program in South Africa conducted by The Centre for HIV and AIDS Prevention Studies (CHAPS) in 2013.
Variable cost data are all sourced from CHAPS 2013, except HIV testing. HIV testing cost data is sourced from the South African government figures obtained from officials by BMGF.

Table S15. Cost assumptions for early ART
Note that we assume the cost of late ART to be $255 per person per year to match early ART costs excluding demand incentives.

Population group	Coverage level	Fixed costs	Variable costs
All	Constant	n/a	n/a
	Medium	$10M / year	$275 / person / year<br>- Commodity: $120<br>- Labs: $53<br>- Outpatient, other: $40<br>- Demand incentives: $20<br>- Salaries: $40
	Maximum	$11.6M / year<br>- additional M&E, mass behaviour change, outreach, HR for FSW	$295 / person / year<br>- Commodity: $120<br>- Labs: $53<br>- Outpatient, other: $40<br>- Demand incentives: $40<br>- Salaries: $40

Fixed costs: Comprised of early ART's portion of mass-media campaigns ($10M, or $1.43M for each of the 7 sub-populations). Assumes no extra above-service delivery costs needed for program management, lab infrastructure.
Variable costs: Unit cost data for commodities, labs, outpatient, and salary data are sourced from South African government officials. Salary costs reflect an assumption that current human resources (HR) infrastructure is at capacity. Reflecting a hypothesis that it may be harder to reach/incentivize relatively heathier and/or higher-risk patients, BMGF added an estimate for demand incentives based on field estimates for transport vouchers for new patients seeking care.
The cost estimates do not include morbidity and mortality benefits.

Table S16. Cost assumptions for oral PrEP

Population group	Coverage level	Fixed costs	Variable costs
Female sex workers	Constant	n/a	- n/a
	Medium	$2.5M one time + $1.67 M per year	$170 per person per year<br>- Commodity: $70<br>- Testing: $40<br>- Salaries: $60
	Maximum	$2.5M one time + $3M per year	$183 per person per year<br>- Commodity: $70<br>- Testing: $40<br>- Salaries: $60<br>- Outreach: $13
High-risk women, 15-30 years	Constant	n/a	- n/a
	Medium	$2.5M one time + $1.67 M per year	$170 per person per year<br>- Commodity: $70<br>- Testing: $40<br>- Salaries: $60
	Maximum	$2.5M one time + $1.67 M per year	$190 per person per year<br>- Commodity: $70<br>- Testing: $40<br>- Salaries: $60<br>- Outreach: $20
Low-risk women, 15-30 years	Constant	n/a	n/a
	Medium	n/a	n/a

	Maximum	$2.5M one time + $1.67 M per year	$190 per person per year
Women, 30-49 years	Constant	n/a	n/a
	Medium	n/a	n/a
	Maximum	$2.5M one time + $1.67 M per year	$190 per person per year
High-risk men, 15-49 years	Constant	n/a	n/a
	Medium	n/a	n/a
	Maximum	$2.5M one time + $1.67 M per year	$170 per person per year
Low-risk men, 15-49 years	Constant	n/a	n/a
	Medium	n/a	n/a
	Maximum	$2.5M one time + $1.67 M per year	$170 per person per year

Fixed costs of introducing launch planning and mass media. Launch planning costs are based on the LIST tool and include market management, policy guidance, WHO pre-qualification, regional and country policy consultations, demand creation, and delivery capacity. Costs are based on historical estimates of previous product launches. We estimate that $15M is needed to launch oral PrEP in South Africa, or $2.5M for each of the 6 sub-groups included in the table above (here we collapse 30-49 year-old women into one group). We estimate an annual cost of $10M for mass media, or $1.67M per year for each of the 6 sub-groups.
Commodity costs are sourced from the South African government tenders for Tenofovir 300mg and Emtricitabine 200mg generic combination (TDF + FTC) with an adjustment of 5% and 10% for inflation and wastage, respectively.
Testing: Testing regime confirmed by WHO as: a) HIV test - initial testing, then every 3 months; b) creatinine test before initiation, then quarterly follow-up, then every 6 months. Test costs sourced from the South African Government at $6/HIV test and $2.5/creatinine test.
Salaries: Sourced from the South African Government

Outreach: For sex workers (only): assumes monthly visits to increase awareness on condoms, PrEP, vaginal rings, and to distribute condoms and lube. 1:1 model. $40 per FSW shared by 3 programs ($13 each). These costs are based on published literature of the Avahan program.[32] Outreach costs for other high-risk women are assumed to be higher as they may be harder to reach.

Table S17. Cost assumptions for IVR.

Population group	Coverage level	Fixed costs	Variable costs
Female sex workers	Constant	n/a	n/a
	Medium	$2M one time + $1M / year	$107 per person per year<br>- Commodity: $70<br>- Labs, HR: $20<br>- Outreach: $17
	Maximum	$2M one time + $1M / year	$115 per person per year<br>- Commodity: $70<br>- Labs, HR: $20<br>- Outreach: $25
High-risk women, 15-30 years	Constant	n/a	n/a
	Medium, Maximum	$2M one time + $1M / year	$115 per person per year
Low-risk women, 15-30 years	Constant	n/a	n/a
	Medium, Maximum	$2M one time + $1M / year	$115 per person per year
High-risk women, 30-49 years	Constant	n/a	n/a
	Medium, Maximum	$2M one time + $1M / year	$115 per person per year
Low-risk women, 30-49 years	Constant	n/a	n/a
	Medium, Maximum	$2M one time + $1M / year	$115 per person per year

Fixed cost: An estimated $10M launch cost divided equally over 5 sub-groups. Launch planning costs are based on the LIST tool and include market management, policy guidance, WHO pre-qualification, regional and country policy consultations, demand creation, and delivery capacity. Costs are based on historical estimates of previous product launches. Mass media is estimated to cost $5M annually, or $1M per year for each of the 5 sub-groups.

Variable costs: Commodity costs are BMGF internal estimates based on internal target product profiles of ongoing research and development (R&D) efforts. The IVR is estimated to cost $5 each, and one ring is used per person per month. Delivery costs include lab and human resource investments and assume only one annual visit and an HIV test at initiation. Outreach costs for sex workers (only) assumes monthly visits to increase awareness on condoms, PrEP, vaginal rings, and to distribute condoms and lube. 1:1 model. $40 per FSW shared by 3 programs ($13 each). These costs are based on published literature of the Avahan program.[32]

Table S18. Cost assumptions for LA ARVs

Population group		Fixed costs	Variable costs
Female sex workers	Constant	n/a	n/a
	Medium	$1.67M one time + $0.83M / year	$180 per person per year<br>- Commodity: $110<br>- Service delivery: $70
	Maximum	$1.67M one time + $0.83M / year	$200 per person per year<br>- Commodity: $110<br>- Service delivery: $70<br>- Demand incentive: $20
High-risk women, 15-30 years	Constant	n/a	n/a
	Medium, Maximum	$1.67M one time + $0.83M / year	$180 per person per year
Low-risk women, 15-30 years	Constant	n/a	n/a
	Medium, Maximum	$1.67M one time + $0.83M / year	$180 per person per year
Women, 30-49 years	Constant	n/a	n/a
	Medium, Maximum	$1.67M one time + $0.83M / year	
High-risk men, 15-49 years	Constant	n/a	n/a
	Medium, Maximum	$1.67M one time + $0.83M / year	$180 per person per year
Low-risk men, 15-49 years	Constant	n/a	n/a
	Medium, Maximum	$1.67M one time + $0.83M / year	$180 per person per year

Fixed costs of introducing launch planning and mass media. An estimated $10M launch cost divided equally over 5 sub groups. Launch planning costs are based on the LIST tool and include market management, policy guidance, WHO pre-qualification, regional and country policy consultations, demand creation, and delivery capacity. Costs are based on historical estimates of previous product launches. Assume $10M for all of South Africa at launch, or $1.7M for each of the 6 sub-groups under LA ARVs in the model (here we collapse 30-49 year-old women into one group). Mass media at $5M total annually, or $0.83M per year for each sub-group.
Variable costs: Commodity costs are BMGF internal estimates based on internal target product profiles of ongoing R&D efforts. Service delivery costs are based on ART costs and assume an HIV test is required at initiation. Like early ART, assumes demand incentives are needed to reach maximum coverage targets.

Table S19. Cost assumptions for bNAbs.

Population group		Fixed costs	Variable costs
Female sex workers	Constant	n/a	n/a
	Medium	$1.7M one time + $0.83M / year	$190 per person per year<br>- Commodity: $110<br>- Delivery: $80
	Maximum	$1.7M one time + $0.83M / year	$210 per person per year<br>- Commodity: $110<br>- Delivery: $80<br>- Demand incentive: $20
High-risk women, 15-30 years	Constant	n/a	n/a
	Medium, Maximum	$1.7M one time + $0.83M / year	$190 per person per year
Low-risk women, 15-30 years	Constant	n/a	n/a
	Medium, Maximum	$1.7M one time + $0.83M / year	$190 per person per year
Women, 30-49 years	Constant	n/a	n/a
	Medium, Maximum	$1.7M one time + $0.83M / year	$190 per person per year
High-risk men, 15-49 years	Constant	n/a	n/a
	Medium, Maximum	$1.7M one time + $0.83M / year	$190 per person per year
Low-risk men, 15-49 years	Constant	n/a	n/a

	Medium, Maximum	$1.7M one time + $0.83M / year	$190 per person per year

Fixed costs of introducing launch planning and mass media. An estimated $10M launch cost divided equally over 5 sub groups. Launch planning costs are based on the LIST tool and include market management, policy guidance, WHO pre-qualification, regional and country policy consultations, demand creation, and delivery capacity. Costs are based on historical estimates of previous product launches. Assume $10M for all of South Africa at launch, or $1.7M for each of the 6 sub-groups under BNAbs in the model (here we collapse 30-49 year-old women into one group). Mass media at $5M total annually, or $0.83M per year for each sub-group.
Variable costs: Commodity costs are BMGF internal estimates based on internal target product profiles of ongoing R&D efforts. Cost assumes that bNAbs would be priced competitively with oral PrEP. Commodity costs are based on 150mg dose every 3 months. Delivery costs are based on oral PrEP estimates and increased to include an estimate for cold chain recurrent costs. Like early ART, assumes demand incentives are needed to reach maximum coverage targets. Costing assumes no need to subsidize drug facility infrastructure costs.

Table S20. Cost assumptions for vaccines.

Population group		Fixed costs	Variable costs
All	Constant	n/a	n/a
	P5-like vaccine during scale-up	$65M one time + $5M per year	Routine: $50 per vaccination initially + $9 boost every 2 years<br>Campaign: $40 per vaccination initially + $7 boost every 2 years
	P5-like vaccine at scale & idealized vaccine	P5-like vaccine: additional $10M per year<br>Idealised vaccine: $5M per year throughout	Routine: $60 per vaccination initially + $9 boost every 2 years<br>Campaign: $50 per vaccination initially + $7 boost every 2 years

Fixed costs assume one-time cost of (1) a $15M investment for all of South Africa at launch, based on the LIST tool and include market management, policy guidance, WHO pre-qualification, regional and country policy consultations, demand creation and delivery capacity, and (2) $50M to support facility infrastructure. These costs are based on historical estimates of previous product launches. Annual mass media costs are added at $5M per year for the whole population.

Variable costs: Commodity costs are BMGF internal estimates based on internal target product profiles of ongoing R&D efforts. Service delivery costs are based on costs from the literature for HPV vaccines and assume $3 per adolescent reached and assume some delivery synergies with HPV vaccines. Delivery costs for adult populations are assumed to be $6 per adult, assuming that delivery is more expensive for adults given separate delivery channel would be needed. Assumes 5 dose presentation (0, 1, 3, 6, 12 months) + booster every 2 years. Initially given to adults, then (2-5 yrs later) to 14 year-olds. Delivery costs assume no HIV tests/labs are needed, and no implications for diagnostic testing.

Figure S8. Univariate intervention impact

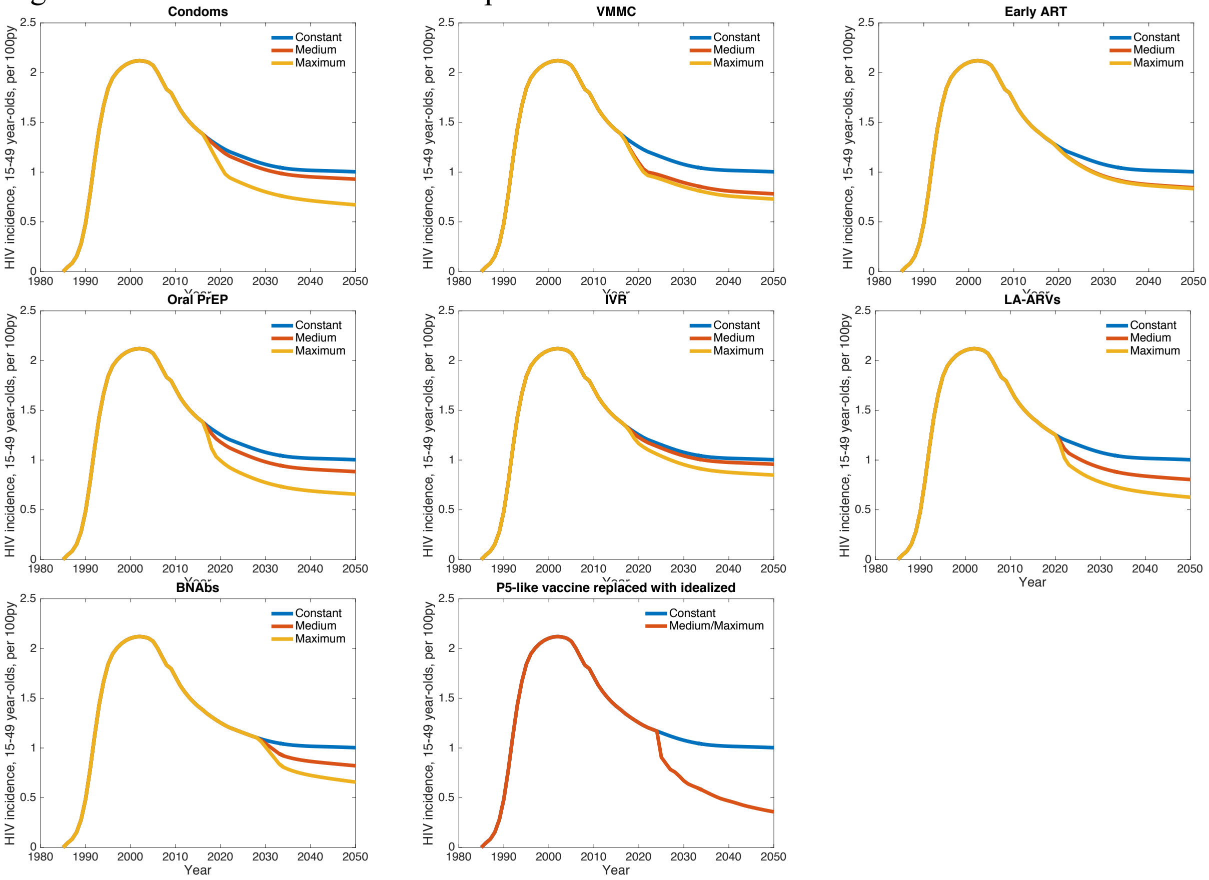

Figure S9. Cost profiles of Constant, Medium, Optimal Allocation and Maximum scenarios, 2015-2050.

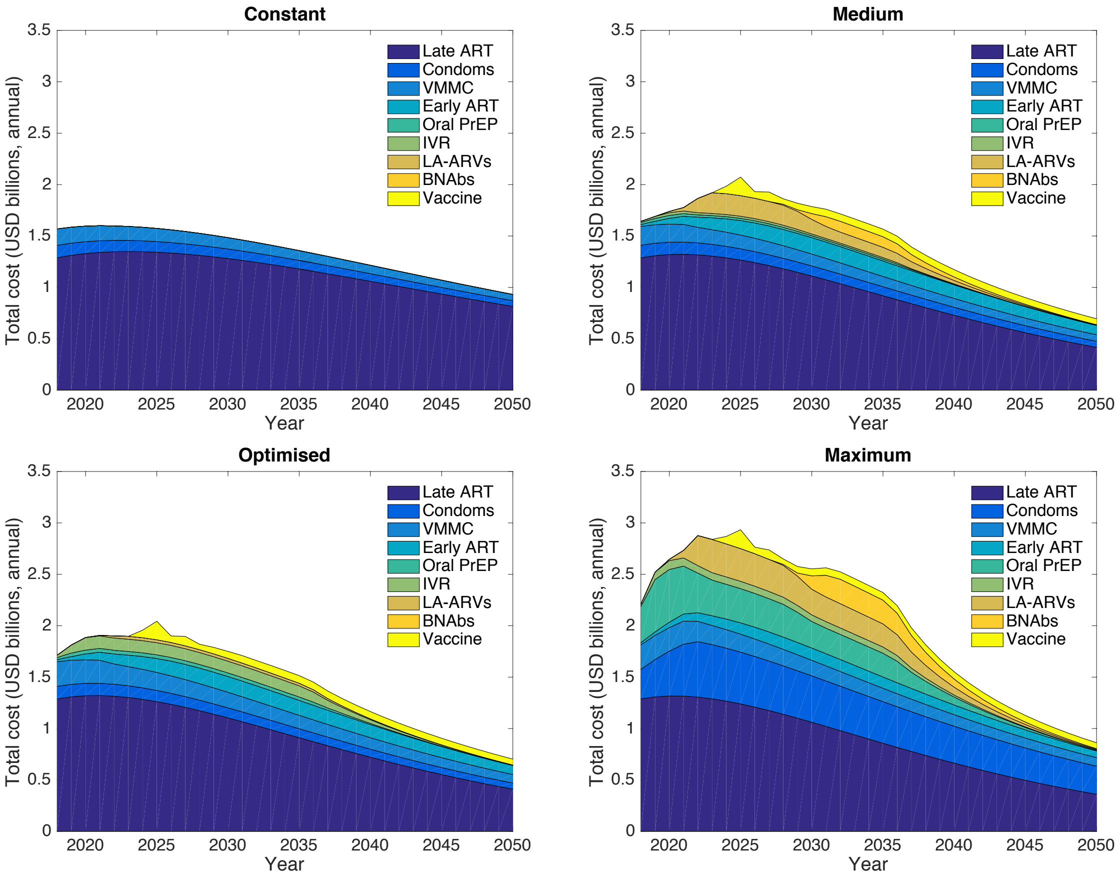

Figure S10. Sensitivity analysis around variable costs of interventions
A multiplicative factor was applied to the variable cost only of each intervention and varied from 0.1 to 1.

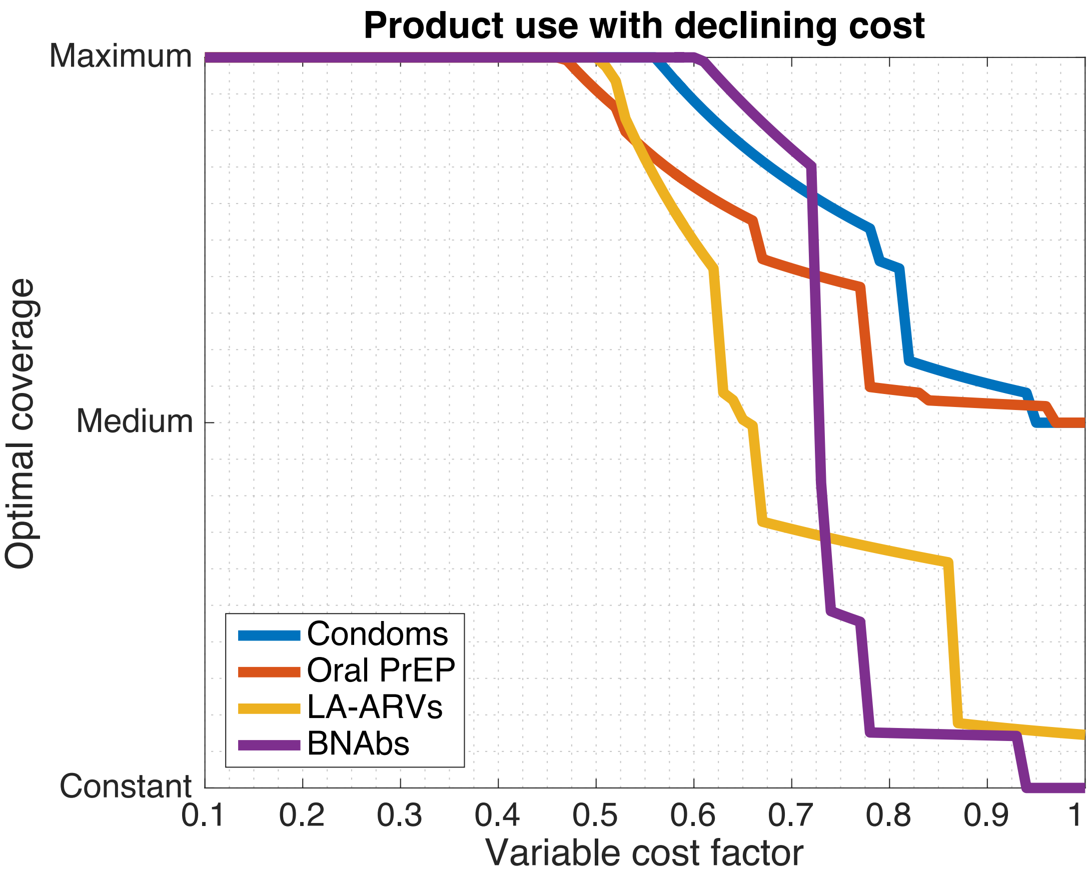

Figure S11. Sensitivity analysis around fixed costs of vaccination
A multiplicative factor was applied to the one-time fixed cost only of a vaccination intervention (baseline: $65M) and varied from 1 to 400.

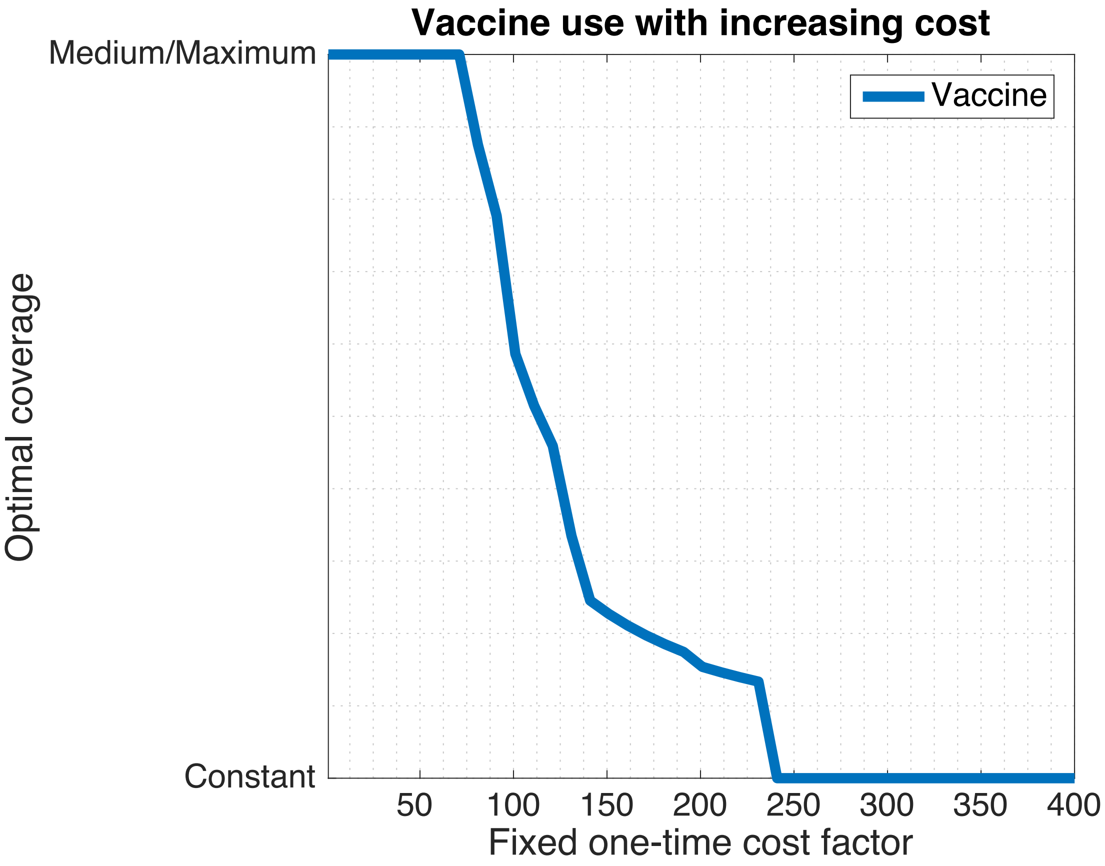

Table S21. Interventions included in the health production function (Figure 1A).

HPF point	Condoms	VMMC	Early ART	Oral PrEP	IVR	LA-ARVs	BNAbs	Vaccine
**1**	Medium	Medium	Constant	Constant	Constant	Constant	Constant	Constant
**2**	Medium	Medium	Constant	Constant	Constant	Constant	Constant	Medium / Maximum
**3**	Medium	Medium	Constant	Medium	Constant	Constant	Constant	Medium / Maximum
**4**	Medium	Medium	Maximum	Medium	Constant	Constant	Constant	Medium / Maximum
**5**	Medium	Maximum	Maximum	Medium	Constant	Constant	Constant	Medium / Maximum
**6**	Medium	Maximum	Maximum	Medium	Maximum	Constant	Constant	Medium / Maximum
**7**	Medium	Maximum	Maximum	Medium	Maximum	Medium	Constant	Medium / Maximum
**8**	Medium	Maximum	Maximum	Maximum	Constant	Maximum	Medium	Medium / Maximum
**9**	Medium	Maximum	Maximum	Maximum	Constant	Maximum	Constant	Medium / Maximum
**10**	Maximum	Maximum	Maximum	Maximum	Constant	Maximum	Medium	Medium / Maximum
**11**	Maximum	Maximum	Maximum	Maximum	Constant	Maximum	Constant	Medium / Maximum
**12**	Maximum	Maximum	Maximum	Maximum	Constant	Maximum	Maximum	Medium / Maximum
**13**	Maximum	Maximum	Maximum	Maximum	Maximum	Maximum	Maximum	Medium / Maximum

HPF points refer to the blue circles in Figure 1A, numbered from the bottom left to the top right of the figure.

Figure S12. Optimal allocation in Cross River, Nigeria

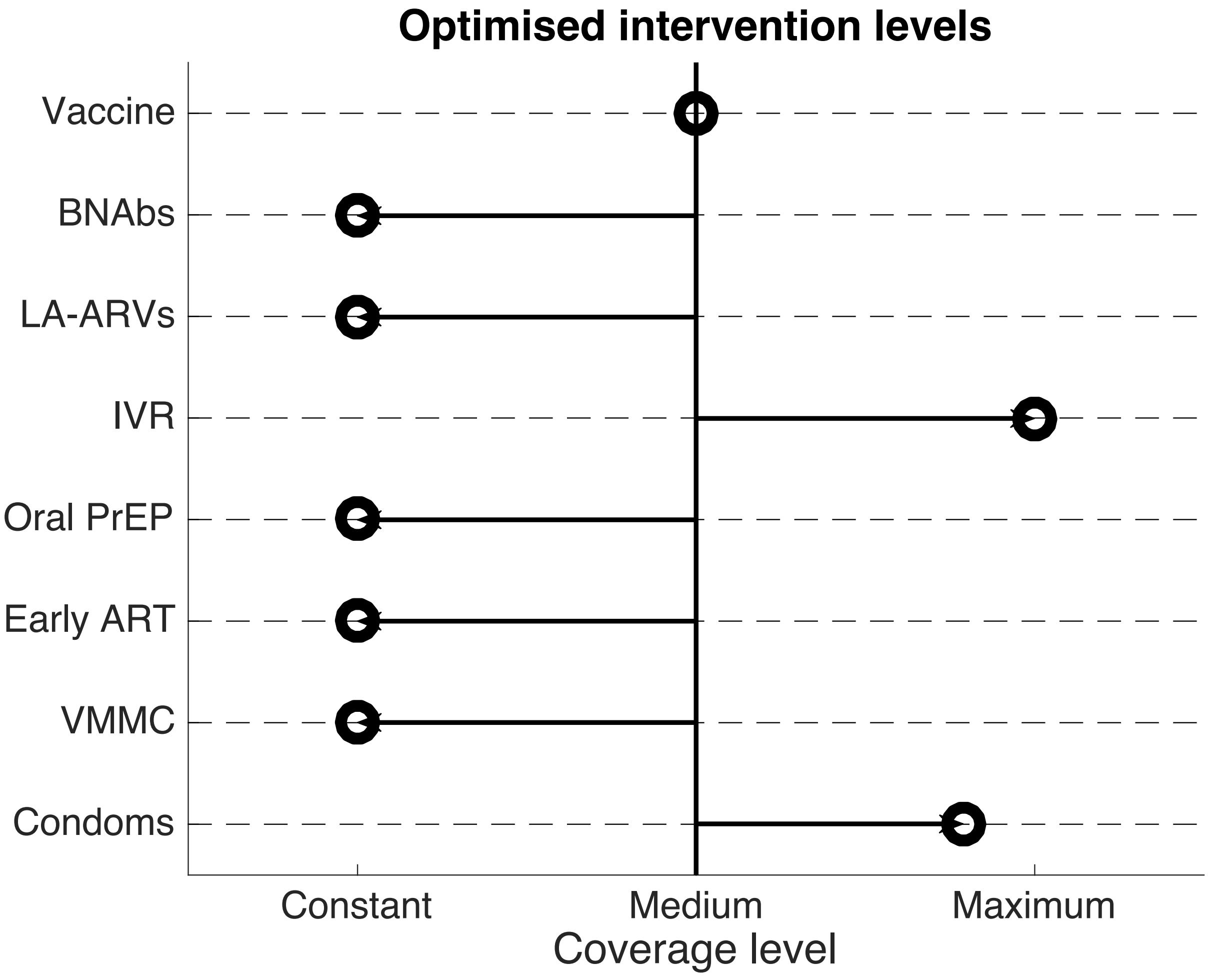

References

1. Anderson R, May R. Infectious diseases of humans: dynamics and control: Oxford; 1991.
2. Garnett G, Anderson R. Factors controlling the spread of HIV in heterosexual communities in developing countries: patterns of mixing between different age and sexual activity classes. *Philosophical Transactions of the Royal Society B: Biological Sciences* 1993; **342**: 137-59.
3. Garnett G, Anderson R. Sexually transmitted diseases and sexual behaviour: insights from mathematical models. *Journal of Infectious Diseases* 1996; **198**: 687-93.
4. Hollingsworth T, Anderson R, Fraser C. HIV-1 transmission by stage of infection. *Journal of Infectious Diseases* 2008; **198**(5): 687-93.
5. Shisana O, Rehle T, Simbayi LC, et al. South African National HIV Prevalence, Incidence and Behaviour Survey, 2012. Cape Town, 2014.
6. Boily M-C, Baggaley R, Wang L, et al. Heterosexual risk of HIV-1 infection per sexual act: systematic review and meta-analysis of observational studies. *The Lancet Infectious Diseases* 2009; **9**(2): 118-29.
7. Donnell D, Baeten J, Kiarie J, et al. Heterosexual HIV-1 transmission after initiation of antiretroviral therapy: a prospective cohort analysis. *The Lancet* 2010; **375**(9731): 2092-8.
8. Johnson L. Access to Antiretroviral Treatment in South Africa, 2004-2011. *South Africa Journal of HIV Medicine* 2012; **13**(1).
9. Cohen MS, Chen YQ, McCauley M, et al. Prevention of HIV-1 Infection with Early Antiretroviral Therapy. *New England Journal of Medicine* 2011; **365**(6): 493-505.
10. Department of Health, Medical Research Council, OrcMacro. South Africa Demographic and Health Survey 2003. Pretoria: Department of Health, 2007.
11. Actuarial Society of South Africa. ASSA2008 Model. 2011.
12. THE NATIONAL HIV AND SYPHILIS PREVALENCE SURVEY SOUTH AFRICA 2007. South Africa: National Department of Health, 2008.
13. Granich RM, Gilks CF, Dye C, De Cock KM, Williams BG. Universal voluntary HIV testing with immediate antiretroviral therapy as a strategy for elimination of HIV transmission: a mathematical model. *The Lancet* 2009; **373**(9657): 48-57.
14. Mossong J, Grapsa E, Tanser F, Bärnighausen T, Newell M-L. Modelling HIV incidence and survival from age-specific seroprevalence after antiretroviral treatment scale-up in rural South Africa. *AIDS (London, England)* 2013; **27**(15): 2471-9.
15. Shisana O, Rehle T, Simbayi L, et al. South African National HIV Prevalence, Incidence, Behaviour and Communication Survey, 2008: A turning tide among teenagers? Cape Town, 2009.
16. Cross River State Agency for the Control of AIDS (CRSACA). Cross River State Social and Behavior Change Communication Survey. Calabar, Nigeria: Cross River State Agency for the Control of AIDS (CRSACA),, 2013.
17. Population Pyramids of the World from 1950 to 2100. http://populationpyramid.net/nigeria/1985/ (accessed April 20 2016).
18. Cross River State Ministry of Health, FHI 360, UNAIDS. Operational Plan for the Elimination of Mother-to-Child Transmission of HIV. Nigeria: Cross River State Ministry of Health, FHI 360, UNAIDS, 2013.
19. Bashorun A, Nguku P, Kawu I, et al. A description of HIV prevalence trends in Nigeria from 2001 to 2010: what is the progress, where is the problem? *The Pan African Medical Journal* 2014; **18**(Suppl 1): 3.
20. Pinkerton SD, Abramson PR. Effectiveness of condoms in preventing HIV transmission. *Social Science & Medicine* 1997; **44**(9): 1303-12.

21. Weller SC, Davis-Beaty K. Condom effectiveness in reducing heterosexual HIV transmission. *Cochrane Database of Systematic Reviews* 2002; **2002**(1).
22. Auvert B, Taljaard D, Lagarde E, Sobngwi-Tambekou J, Sitta R, Puren A. Randomized, Controlled Intervention Trial of Male Circumcision for Reduction of HIV Infection Risk: The ANRS 1265 Trial. *PLoS Med* 2005; **2**(11): e298.
23. Bailey RC, Moses S, Parker CB, et al. Male circumcision for HIV prevention in young men in Kisumu, Kenya: a randomised controlled trial. *The Lancet* 2007; **369**(9562): 643-56.
24. Gray RH, Kigozi G, Serwadda D, et al. Male circumcision for HIV prevention in men in Rakai, Uganda: a randomised trial. *The Lancet* 2007; **369**(9562): 657-66.
25. Donnell D, Baeten JM, Bumpus NN, et al. HIV Protective Efficacy and Correlates of Tenofovir Blood Concentrations in a Clinical Trial of PrEP for HIV Prevention. *JAIDS Journal of Acquired Immune Deficiency Syndromes* 2014; **66**(3): 340-8.
26. Nel A, Kapiga S, Bekker L-G, Devlin B, Borremans M, Rosenberg Z. Safety and Efficacy of Dapivirine Vaginal Ring for HIV-1 Prevention in African Women. Conference on Retroviruses and Opportunistic Infections. Boston; 2016.
27. Kovarova M, Swanson MD, Sanchez RI, et al. A long-acting formulation of the integrase inhibitor raltegravir protects humanized BLT mice from repeated high-dose vaginal HIV challenges. *Journal of Antimicrobial Chemotherapy* 2016.
28. Margolis DA, González-García J, Stellbrink H-J, et al. Cabotegravir+Rilpivirine as Long-Acting Maintenance Therapy: LATTE-2 Week 32 Results. Conference on Opportunistic Infections and Retroviruses. Boston, Massachusetts; 2016.
29. Caskey M, Klein F, Lorenzi JCC, et al. Viraemia suppressed in HIV-1-infected humans by broadly neutralizing antibody 3BNC117. *Nature* 2015; **522**(7557): 487-91.
30. Hughes JP, Baeten JM, Lingappa JR, et al. Determinants of Per-Coital-Act HIV-1 Infectivity Among African HIV-1–Serodiscordant Couples. *Journal of Infectious Diseases* 2012; **205**(3): 358-65.
31. Global HIV Vaccine Enterprise. P5 Partnership. 2015. http://www.vaccineenterprise.org/content/P5Partnership (accessed 4th January 2015).
32. Chandrashekar S, Guinness L, Pickles M, et al. The Costs of Scaling Up HIV Prevention for High Risk Groups: Lessons Learned from the *Avahan* Programme in India. *PLoS ONE* 2014; **9**(9): e106582.
